# Recent Development on Narrow Bandgap Conjugated Polymers for Polymer Solar Cells

**DOI:** 10.3390/polym9020039

**Published:** 2017-01-28

**Authors:** Yueyue Gao, Ming Liu, Yong Zhang, Zhitian Liu, Yulin Yang, Liancheng Zhao

**Affiliations:** 1 School of Materials Science and Engineering, Harbin Institute of Technology, Harbin 150001, China; gaoyueyuewang@126.com (Y.G.); mingliu016@163.com (M.L.); lczhao@hit.edu.cn (L.Z.); 2 MIIT Key Laboratory of Critical Materials Technology for New Energy Conversion and Storage, School of Chemistry and Chemical Engineering, Harbin Institute of Technology, Harbin 150001, China; ylyang@hit.edu.cn; 3 College of Materials Science and Engineering, Wuhan Institute of Technology, Wuhan 430205, China; able.ztliu@gmail.com

**Keywords:** conjugated polymers, narrow bandgap, polymer solar cells, photovoltaic cells

## Abstract

There have been exciting developments in the field of polymer solar cells (PSCs) as the potential competitor to the traditional silicon-based solar cells in the past decades. The most successful PSCs are based on the bulk hetero-junction (BHJ) structure, which contains a bicontinuous nanoscale interpenetrating network of a conjugated polymer and a fullerene blend. The power conversion efficiencies (PCEs) of BHJ PSCs have now exceeded 11%. In this review, we present an overview of recent emerging developments of narrow bandgap conjugated polymers for PSCs. We focus on a few important acceptors used in the donor-acceptor type conjugated polymers for highly efficient PSCs. We also reviewed the emerged donor-π-acceptor (D-π-A) side chains polymers. The band-gaps and energy levels as well as the photovoltaic performances of conjugated polymers are discussed.

## 1. Introduction

Solar power is the most abundant renewable energy source. Power as high as 120,000 Tw from sunlight reaches the Earth’s surface every year and this is significantly beyond the whole world’s energy demand (15 Tw/year) [1]. In the past decades, there have been many energy productions through utilizing the sunlight, for example the conventional silicon solar cells. Another alternative option that has emerged in the past years is organic photovoltaic cells, including dye-sensitized solar cells (DSSCs) [2], polymer solar cells (PSCs) [3,4,5,6,7], and small-molecule solar cells [8]. Among these, polymer solar cells based on bulk hetero-junction (BHJ) structure are of significant attraction because of the low-cost, light-weight, flexibility and the potential fabrication through high-throughput and large-area roll-to-roll processing method [9]. The BHJ structure is commonly formed by blending an electron-rich conjugated polymer as donor and an electron-deficient fullerene as acceptor with bi-continuous nanoscale interpenetrating network, in which both donor and acceptor materials are physically mixed to create a large interface area and partial phase segregation throughout the whole active layer because of the different surface energy of both materials. The first PSC based on BHJ structure used MEH-PPV as the electron-rich donor and C_60_ derivatives as the electron-deficient acceptor, and was processed by spin-coating from the mixture solution of two materials. The efficiency was rather low at that time. Currently, BHJ polymer solar cells reach PCEs of more than 11% in simulated AM 1.5 G light throughout the optimization of both materials and devices engineering [10].

**Figure 1:**
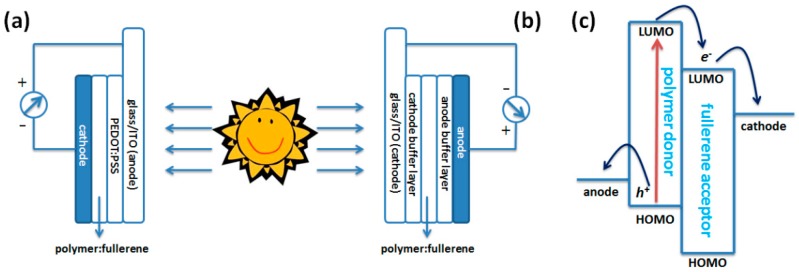
The schematic representation of conventional (**a**) and inverted structures (**b**) and the schematic energy diagram of the sandwiched bulk hetero-junction (BHJ) cell (**c**).

There are two kinds of device structures commonly used for BHJ polymer solar cells. One is the conventional structure and another is the inverted structure. In a conventional device (Figure 1a), indium tin oxide (ITO) is often used as the anode for collecting holes, and the low work function materials, such as Ca and Al, are used as the cathode for the collection of electron. Instead, in inverted device (Figure 1b), ITO with a buffer layer, such as TiO_x_ and ZnO, is commonly used as the cathode electrode [11,12,13,14]. And the anode could be the high work-function metals (Ag or Au) with an anode buffer layer, i.e., PEDOT:PSS [15]. With either conventional or inverted structure, the operational principle is same as shown in Figure 1c. When the BHJ film made up of polymer and fullerene is illuminated under sunlight, excitons are generated and mainly came from the polymer because of the strong absorption overlapping with sunlight spectrum and small band-gap energy for excitation. The exciton actually exists as Coulombical bounded electron-hole pair in polymer phase because of the low dielectric constant of conjugated polymers. The formed excitons will diffuse to the interface of polymer and fullerene, in which exciton dissociation could happen when the electron transfers to the LUMO energy level of the fullerene and the hole is kept on the HOMO energy level of polymer. After that, the electron and hole can diffuse to the corresponding electrode, therefore, to generate the current [16,17]. In fact, the whole process is complicated and affected by several factors, such as charge separation efficiency, charge recombination and nano-scale phase separation between polymer and fullerene, and so on [18].

There are three important parameters to characterize a cell, and they are open circuit voltage (*V*_oc_), short circuit current density (*J*_sc_) and fill factor (FF). The overall performance PCE is calculated in the following equation:

$$\mathrm{PCE}\left(\%\right)=\frac{V_{\mathrm{oc}}\times J_{\mathrm{sc}}\times FF}{P_{\mathrm{in}}}\times 100\%$$

**Figure 2:**
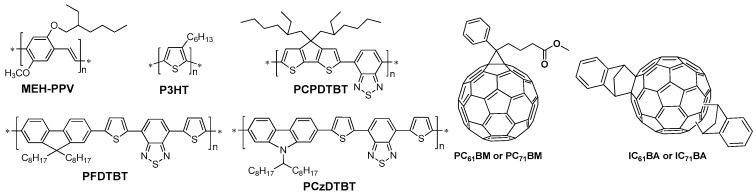
The chemical structures of some classical conjugated polymers and fullerene derivatives for BHJ cells.

In the equation, *P*_in_ is the power density of the incident light intensity (i.e., 100 mW/cm^2^ under simulated AM1.5G illumination) [16,17]. It is known that the *V*_oc_ of PSCs is mainly limited by the difference between the highest occupied molecular orbital (HOMO) of the polymer donor and the lowest unoccupied molecular orbital (LUMO) of the fullerene acceptor, although some electrode engineering could also affect the *V*_oc_ [19,20,21,22]. The *J*_sc_ of PSCs is directly related to the band-gap of conjugated polymer and efficient charge separation, etc. The dependence of FF is more complex and can be optimized through the device engineering. It clearly shows that the polymer materials play a decisive role on achieving high PCE, although the careful device optimization is still needed. Over the past decade, researchers have developed many conjugated polymers and fullerene derivatives for the use in BHJ cells [5,6]. Figure 2 lists some classical conjugated polymers and phenyl-C_61_-butyric acid methyl ester (PC_61_BM) and phenyl-C_71_-butyric acid methyl ester (PC_71_BM) for BHJ PSCs. Among all polymers that have been developed, P3HT is one of the most widely used and the PCEs of 5% have been reported [23,24]. However, the relatively large band-gap (2.0 eV) and high HOMO energy level (−5.0 eV) limit the possibility to further improve the device performance based on P3HT [24,25]. In 2007, Heeger’s group reported the polymer PCPDTBT (Figure 2), which was a kind of donor-acceptor conjugated polymer with a lower band-gap (1.46 eV). The PCE can reach 5.5% in the optimized device [26]. Although the improved *J*_sc_ was achieved, the *V*_oc_ was still much low. Then, Lecerlc et al. synthesized a polymer PCzDTBT (Figure 2), which had a low-lying HOMO energy level. After a series of device engineering, the PCE of 5%–6% could be achieved with an improved *V*_oc_, which represented the best efficiency at that time. To try to reach a higher PCE, great effort had been made by many research groups by focusing on the novel donor-acceptor polymers in the past years. Regarding the fullerene derivatives, in the past decades PCBM was still a commonly electron acceptor as it can be widely used with various polymers. However, the syntheses of novel non-fullerene derivatives have been emerged and the record-high efficiencies have been achieved [27,28,29,30,31].

Regarding the synthesis of D-A conjugated polymer, besides the traditional Suzuki or Stille polymerizations, synthesizing such polymers by a green chemistry method has received more and more attention. For example, Lipomi and coworkers reported the strategies for synthesizing conjugated polymers using green chemistry. The main idea of green chemistry is to synthesize a polymer with low energy intensity, minimal production of toxic waste and low cost [32]. Most important, Kanbara and coworkers also reported that the green chemistry method could proceed with a reduced amount of catalysts with short reaction time compared with the conventional polycondensation based on cross-coupling reactions [33,34]. Recently, Vaccaro and coworkers summarized the methods to synthesize such polymers using green chemistry [35,36]. Even though green chemistry promises a bright future, the current application is still limited and a great effort is needed to improve it in order to have wider application.

The rapid developments of conjugated polymers for polymer solar cells have emerged as one of the main research areas in polymer chemistry. So, to better understand the current status of such development, it becomes highly necessary to have a summary about the progress of the current state-of-art conjugated polymers for highly efficient PSCs. In this review, we will give the broad introduction of the developments of conjugated polymers in PSCs by summarizing a few important acceptors and their advances, which will receive a broad interest for the researchers to understand the photovoltaic conjugated polymers, instead of focusing on a specific type conjugated polymer [37,38,39]. We will mainly focus on recent rapid advances in the development of conjugated polymers and some key parameters for polymer design. The strategies for further development and improvement on narrow bandgap conjugated polymers are discussed with outlooks for future research.

## 2. Conjugated Polymers

### 2.1. Thieno[3,4-b]thiophene-Based Polymers

Before being used as the electron-donor materials in PSCs, various poly(thieno[3,4-*b*]thiophene) and its derivatives have been synthesized, but they were mainly applied in the electrochromic devices as a transparent ion-storage layer [40] and in polymer light-emitting diodes as a hole injection layer [41], as well as in the photo-detectors and so on [42]. That is because of their narrow band-gap (less than 1.2 eV) and superior electrical properties.

**Figure 3:**
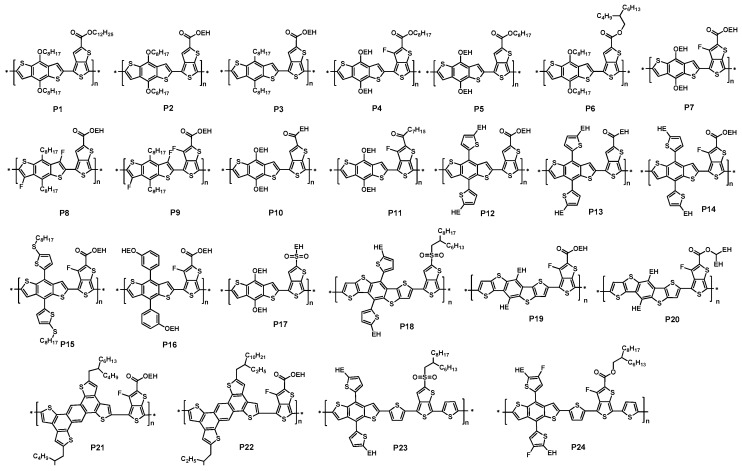
The chemical structures of the thieno[3,4-*b*]thiophene-based conjugated polymers.

A major breakthrough that incorporated the thieno[3,4-*b*]thiophene into a donor-acceptor type conjugated polymer for high efficient PSC was accomplished by Yu and coworkers in 2009 [43]. They synthesized a polymer P1 (Figure 3) containing a benzodithiophene as the donor and a thieno[3,4-*b*]thiophene as the acceptor, which was synthesized via the Stille polycondensation. The solid film of P1 showed an absorption maximum at around 690 nm with the onset at 784 nm. The optical band gap was calculated to be 1.62 eV. The HOMO and LUMO energy levels were measured to be −4.90 eV and −3.20 eV, respectively. The photovoltaic properties of P1 were then investigated with the structure of ITO/PEDOT:PSS/P1:fullerene/Ca/Al. When the PC_61_BM was used as electron acceptor, the PCE of 4.76% was achieved with a *V*_oc_ of 0.58 V, a *J*_sc_ of 12.5 mA/cm^2^ and a FF of 65.4% (Table 1). Further, when PC_71_BM was used as the electron acceptor, an impressive PCE of 5.30% with an improved *J*_sc_ of 15.0 mA/cm^2^, a *V*_oc_ of 0.56 V and a FF of 63.3% was obtained as benefiting from the higher absorption coefficient in the visible region of PC_71_BM.

Later, Yu and coworkers further developed a series of polymers similar to P1 through fine tuning polymer structure either on side chain length or substitutes (P2–P7, Figure 3) [44]. They elaborated three routes to tune the polymer physical properties. First, the polymer side chains in both benzodithiophene and thieno[3,4-*b*]thiophene units were changed to either branched or longer side chains. Through the side chain engineering, the solubility of polymers and the miscibility of polymer with fullerene could be tuned. Second, the substitution of alkoxy side chains in the benzodithiophene donor unit (P2) was changed to the less electron-donating alkyl chains (P3). Third, an electron-withdrawing fluorine atom, which had similar size with hydrogen atom, was used to replace the hydrogen atom in the thieno[3,4-*b*]thiophene unit (P4 and P7 vs. P5). The latter two fine tunes in the polymer backbone reduced the HOMO energy levels of polymers, which had a positive effect on the *V*_oc_ of PSCs.

It was interesting that the electro-optical properties of these polymers were very similar to one another (P1 to P7) with only a slight change on the absorption peak and onset. The optical band-gaps of these polymers were between 1.58 and 1.63 eV. As noted above, the HOMO energy level of the polymer could be lowered by either using a less electron-rich group or introducing an electron-withdrawing group with directly linking to the polymer backbone. The HOMO energy level of P3 was changed to −5.04 from −4.94 eV of P2, because of the replacement of an alkoxyl side chain with an alkyl side chain in the benzodithiophene unit of P3 while keeping the same chain in thieno[3,4-*b*]thiophene unit. Further, the introduction the fluorine atom in the thieno[3,4-*b*]thiophene unit also lowered the HOMO energy level. P4 and P7, which had the fluorine substitute, showed a significant lower HOMO energy level (−5.14 eV for P4 and −5.15 eV for P7) compared with that of P5 (−5.01 eV).

The photovoltaic properties of these polymers were investigated with the same configuration of ITO/PEDOT:PSS/Polymer:PC_61_BM/Ca/Al. Through the devices comparison of P2 with P3, it was clear that the alkyl and alkyoxyl side chains both had an effect on photovoltaic property. The solar device based on P2 showed a *V*_oc_ of 0.60 V, a *J*_sc_ of 12.8 mA/cm^2^ and a FF of 66.3% with an overall PCE of 5.10% (Table 1). As a contrast, an improved *V*_oc_ of 0.74 V was achieved in the device based on P3. This was because of its low-lying HOMO energy level. Combined with a *J*_sc_ of 13.1 mA/cm^2^ and a FF of 56.8%, the PCE of P3 device increased to 5.53% mainly due to the enhancement on the *V*_oc_. However, in the case of P4 and P5 devices for the comparison of fluorine atom’s effect, the moderate and similar PCE (3%) were achieved, but an increased *V*_oc_ (0.76 V) in P4 device was found compared with P5 device (0.68 V). It was noted that the moderate performance came from the polymer/PC_61_BM blend. Under the same device condition with PC_71_BM as the electron acceptor, P7, which was a very similar polymer with P4, gave an impressive PCE of 5.63% with a *V*_oc_ of 0.76 V, a *J*_sc_ of 13.58 mA/cm^2^ and a FF of 54.51% (Table 1) [45]. This clearly proved that introduction of fluorine atom provided an effective way to enhance the performance by enhancing the *V*_oc_ of devices.

It was well known that the performances of PSCs largely depended on the morphology of BHJ layer. The continuous interpenetrating networks with small domains were feasible for achieving high performance [16,46]. To this end, transmission electron microscopy (TEM) was used to investigate the morphologies of these polymer/PC_61_BM blend films. The TEM pictures of P2/PC_61_BM blend film were found to have the finer features than that of P1/PC_61_BM film due to the increased miscibility of P2 and PC_61_BM when shortening dodecyl side chain (P1) to 2-ethylhexyl side chain (P2) in thieno[3,4-*b*]thiophene unit. The improved morphology can be attributed to the improved performance in P2/PC_61_BM device. Further, in the comparison of P2 and P3 that had similar side chain patterns, a few larger features (about 50 nm) in the TEM images of P3/PC_61_BM blend film compared with that of P2 blend film were found. It was believed to be the better packing ability in P3, which reduced its miscibility with PC_61_BM. Although the morphology of P3 was not optimized, P3-based solar cell still showed a PCE of 5.53%, mainly due to the higher *V*_oc_. In P4 and P5 case, large domains were observed in both P4/PC_61_BM and P5/PC_61_BM blend films. This should be ascribed to the reduced the miscibility of polymers with PC_61_BM after changing to the bulky 2-ethylhexyl side chains in benzodithiophene unit. That leaded to large phase separation between polymer chains and PC_61_BM, which reduced the interfacial areas for charge separation. Therefore, the PCE of P4 and P5 were found to be only about 3%. It was interesting that P7, which had a much large bulky 2-ethylhexyl side chains compared with P4, showed a significant improvement on the performance under same device condition, when blended with PC_71_BM instead of PC_61_BM. Although no TEM was reported, it was not easy to say that there were large domains in P7/PC_71_BM blend film. The PCE of a device based on P7 was obtained as high as 6.22%. It was speculated that the high molecular weight of P7 (*M*_w_ = 97.5 kDa, PDI = 2.1) may play an important role on the large difference between P4 (*M*_w_ = 19.3 kDa, PDI = 1.32) and P7 [44,45].

**Figure 4:**
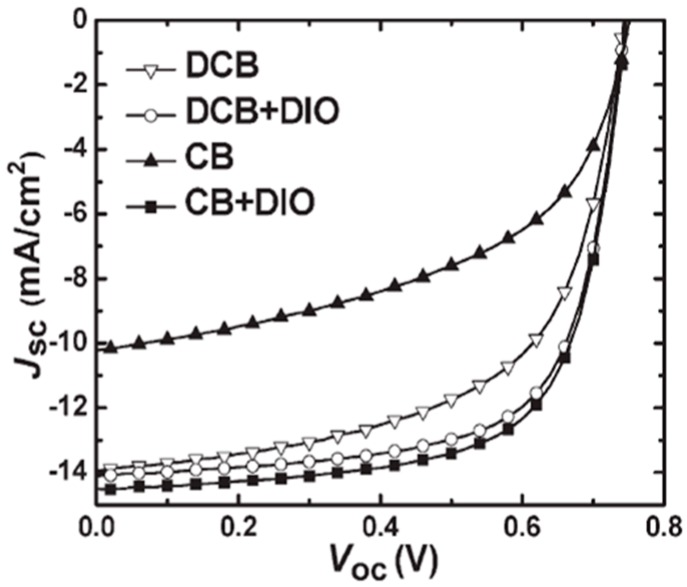
The *J*–*V* curves of P7/PC_71_BM device with different processing solvents (i) DCB only; (ii) DCB with 3% DIO; (iii) CB; and (iv) CB with 3% DIO as solvents. Reproduced with permission from Reference [45], Copyright 2010, Wiley.

It was well known that the morphology could be altered by adding the solvent additive, such as 1,8-diiodooctane (DIO), which could alleviate the aggregation process of polymer and PCBM so as to avoid forming the unfeasible large domain sizes in polymer/PCBM blend film [47]. When 3 vol % of DIO in *o*-dichlorobenzene solution was employed, the morphology of P3/PC_61_BM blend film could be improved [44]. Similar morphology improvement was also observed in P4/PC_61_BM and P5/PC_61_BM blend films. As a result, the PCEs of P3, P4 and P5 all showed an enhancement. The PCE of P3 solar cell reached 5.85%. The P4/PC_61_BM cell showed a dramatic improved PCE to 5.90% from 3.10%. Similar enhancement was also reported in the P7/PC_71_BM blend film [45]. The device with P7/PC_71_BM (1:1.5 *w/w*) film processed from pure *o*-DCB solution showed a maximum PCE of 6.22% with a *V*_oc_ of 0.74 V, a *J*_sc_ of 13.95 mA/cm^2^ and a FF of 60.25% (Table 1). Further study showed that the P7/PC_71_BM-blend film prepared from *o*-DCB/DIO (97/3, *v/v*) gave an increased PCE of up to 7.18% from 6.22% with the large enhancement FF from 60.25% to 68.9%. The *J*_sc_ showed a small change with only increment of 0.14 mA/cm^2^. It was much of interest that the P7/PC_71_BM cell only gave a moderate PCE of 3.92% when processing from chlorobenzene solution with a significant loss of *J*_sc_ and FF. That phenomenon was ascribed to the solvent, which affected the polymer packing. Surprisingly, when DIO was added into the chlorobenzene solution, an unexpected enhancement happened. The *J*_sc_ of the resulting P7/PC_71_BM cell was significantly increased to 14.5 mA/cm^2^ from 10.20 mA/cm^2^. The FF also increased to 69%. Thus, the overall PCE could reach a higher number of 7.40%, as expected. The *J*–*V* curves of P7/PC_71_BM devices with different processing conditions were shown in Figure 4. The dramatic enhancement was attributed to the improved morphology as revealed by the TEM.

**Figure 5:**
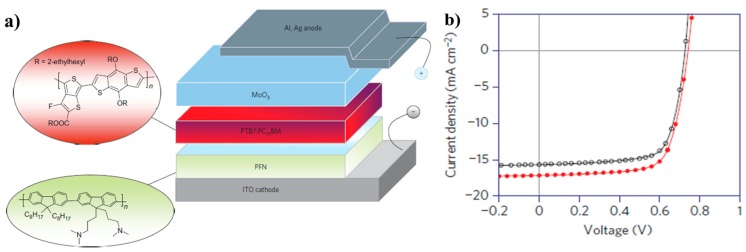
(**a**) Schematic of the inverted-type PSCs, in which the photoactive layer is sandwiched between a PFN-modified ITO cathode and an Al, Ag-based top anode. Insets: chemical structures of the water-/alcohol-soluble conjugated polymer and electron donor materials used in the study. PFN, poly[(9,9-bis(3′-(*N*,*N*-dimethylamino)propyl)-2,7-fluorene)-*alt*-2,7-(9,9-ioctylfluorene)]; PTB7(P7): thie-no[3,4-*b*]thiophene/benzodithiophene; (**b**) Current density versus voltage (*J*–*V*) characteristics under 1000 Wm^2^ AM 1.5G illumination for conventional (open symbols) and inverted (filled symbols) devices. Reproduced with permission from Reference [48], Copyright 2012, Nature publishing group.

In 2012, Wu group reported a highly efficient polymer solar cell by using P7 and PC_71_BM as the active layer with an inverted device structure, they applied an alcohol-/water-soluble conjugated polymer, poly [(9,9-bis(3′-(*N*,*N*-dimethylamino)propyl)-2,7-fluorene)-*alt*-2,7-(9,9-dioctylfluorene)] (PFN) as the ITO surface modifier (Figure 5a). Through careful processing engineering optimizations, a certified PCE of as high as 9.2% was obtained with a remarkable *J*_sc_ of 17 mA/cm^2^ (Figure 5b), *V*_oc_ of 0.75 V and FF of 70% (Table 1), which was the highest reported efficiency in literature at that time. The most important result was that a further increase of 10% in *J*_sc_ could be obtained if the optical constants of the interlayer were matched with those of the stacks of other layers [48].

It was of interest that those polymers containing fluorine substitute showed high performance in PSCs. One of the reasons for the enhanced efficiency was the high *V*_oc_ due to the reduced HOMO energy level. DFT calculation revealed that the HOMO energy level was more affected by the donor unit in the donor-acceptor conjugated polymers. If the electron-withdrawing group (i.e., fluorine) was introduced into the donor unit, the HOMO energy level will be further lower compared with the same substitute in the acceptor unit. Thus, a higher *V*_oc_ following a higher PCE will be anticipated. More recently, the effect of fluorination of polythienothiophene-*co*-benzodithiophenes on the photovoltaic properties was investigated [49]. The difluoro-substituted benzodithiophene was synthesized via multi-steps from benzodithiophene. Then, the corresponding fluorinated polymers P8 and P9 were synthesized by Stile polymerization. The maximum absorption peak and onset of P8 and P9 were both blue-shift compared with P1 and P7, which lead to a slight larger band-gap of 1.75 and 1.73 eV for P8 and P9, respectively. The HOMO and LUMO levels of P8 and P9 were −5.41 and −5.48 eV; −3.60 and −3.59 eV, respectively. These results showed that the introduction of electron-withdrawing group in the donor unit provided a larger effect on both HOMO and LUMO energy levels than that in the acceptor unit. The photovoltaic properties of these polymers were studied with structures of ITO/PEDOT:PSS/polymer:PC_71_BM/Ca/Al. The active layers were spin-coated from *o*-DCB solutions, and the optimized weight ratios of polymer to PC_71_BM were 1:1.5 for both P8 and P9, respectively. The PCEs of P8 and P9-based devices were 3.2% and 2.7% (Table 1), respectively, with a significant loss on *J*_sc_ and FF compared with that of P9 and P7 under same conditions. Further improvement by the addition of DIO was not observed, and the devices showed similar or even worse performance. In addition, the expected enhancement on *V*_oc_ was also not observed. The infeasible morphologies of P8/PC_71_BM and P9 /PC_71_BM blend films were the major reason for the low performance. TEM images of P8 and P9 blend films revealed that there were spherical domains with a size of 50–200 nm, indicating large-scale phase separation and no bicontinuous networks in the blend films. This was attributed to the lower miscibility between polymer and PC_71_BM and the fluorophobicity for PC_71_BM molecules induced by the fluorinated backbones. The loss of photocurrent mainly came from the excitons recombination before reaching the interface of polymer and PC_71_BM in the large spherical domains, which limited the diffusion length of excitons (10 nm). Also, the induced nonuniform electric field also played a role on the decreased *J*_sc_ and FF. To further optimize the device performance, it was suggested to use the PC_71_BM derivative, which has better miscibility with the fluorinated polymers.

It is commonly known that conjugated polymers with alkoxy substitutes usually exhibit higher-lying HOMO energy levels than their alkyl-substituted counterparts. The reason for this is that the alkoxy groups have a much stronger electron donating effect than alkyl ones. Therefore, it was possible to give further reduced HOMO energy level of the above polymer if the ester group was changed to the alkyl ketone substitute in the thieno[3,4-*b*]thiophene unit. With the aim of increasing the *V*_oc_, which was a limit factor for pushing the PCE to higher value, Hou and coworkers synthesized a polymer P10 (Figure 3) with an alkyl ketone-substituted thieno[3,4-*b*]thiophene as the acceptor and the benzodithiophene unit as the donor unit [50]. The P10 showed a band-gap of 1.61 eV, which was similar with the one of P1-P7, indicating that the alkyl ketone substitute only gave a slight effect on optical properties. The HOMO energy level of P10 was −5.12 eV, which was 0.18 eV lower than P2’s HOMO energy level and was in line with the assumption. Photovoltaic test of P10/PC_71_BM solar cell exhibited a *V*_oc_ of 0.70 V, a 0.12 V higher than that of P1/PC_71_BM device. The highest PCE from the optimized devices was 6.58% with a *J*_sc_ of 14.7 mA/cm^2^ and a FF of 64% (Table 1). Further, the same strategy of introducing fluorine atom into the alkyl ketone-substituted thieno[3,4-*b*]thiophene unit was also applied by the same group [51]. Compared with P5 and P10, P11 had a similar absorption spectrum, and also showed a similar band-gap with P10. With fluorine substitutes, the HOMO energy level of P11 was reduced to −5.22 eV and the LUMO energy level (−3.45 eV) was also lowered compared to that of P1–P7 and P10. The BHJ solar cell based on P11 was fabricated at the optimized weight ratio of polymer to PC_71_BM of 1:1.5, a significant increase in *V*_oc_ was clearly showed compared with that of P1 and P10-based devices. The *V*_oc_ could reach 0.76 V. As benefiting from the higher *V*_oc_ along with high *J*_sc_ and FF, the highest PCE of 7.73% (Table 1) was achieved with an average value of 7.40%. On the contrary, P5 and P10 obtained the slightly low PCEs of 5.15% and 6.58% under the same condition. It was noted that the value was achieved from the o-DCB/DIO solution. The device based on P11 was also certified by the National Renewable Energy Laboratory (NREL) with a PCE of 6.77%.

The high PCEs of these thieno[3,4-*b*]thiophene polymers-based solar cells came from the following factors. Firstly, the low band-gaps of these polymers (1.58–1.68 eV) ensured the broad overlapping with sunlight spectrum. The large difference (>0.4 eV) between the LUMO energy levels of the polymer and PCBM (−4.3 eV) provided an enough driving force for excitons dissociation in the donor-acceptor interfaces. Both factors contributed to the high *J*_sc_. Second, the thieno[3,4-*b*]thiophene unit could stabilize the quinoidal structure of polymer backbone. The electron-withdrawing substitute (ester, ketone and fluorine) in thieno[3,4-*b*]thiophene unit also rendered the polymer’s oxidative stable. Further, the polymer backbone became more rigid by combining with planar benzodithiophene unit. The rigidity and planarity of the polymer backbone were directly responsible for the high SCLC mobility (2–6) × 10^−4^ cm^2^·V^−1^·s^−1^ of these polymers. Through selecting the proper processing solvent, the preferred interpenetrating network morphology of these polymer/PCBM blend system could be realized, which could contribute to the efficient charge separation and the charge transport so as to get high FF. In addition, the polymer chains were stacked on the substrate in the face-down conformation as revealed by the Grazing incidence X-ray scattering (GIXS) study.

Further developments on thienothiophene-based polymers were recently conducted by Hou and coworkers [52]. By simply replaced the alkoxy groups with alkylthienyl groups in the benzodithiophene unit, two polymers, P12 and P13 (Figure 3), were synthesized [52]. P13 possessed an alkyl ketone group on the thieno[3,4-*b*]thiophene part, while an ester group was hung on the thieno[3,4-*b*]thiophene unit of P12. These two polymers exhibited similar optical properties. The band-gaps were both 1.60 eV. The HOMO and LUMO energy levels also kept the same, which were around −5.10 and −3.22 eV, respectively. When solar cells were fabricated with PC_71_BM as the acceptor, the P12/PC_71_BM device showed a PCE of 6.21% with a *V*_oc_ of 0.68 V, a *J*_sc_ of 14.59 mA/cm^2^ and a FF of 62.6%. On the contrast, P13 got a slightly higher PCE of 7.59% with the enhancement *V*_oc_ of 0.74 V, a *J*_sc_ of 17.84 mA/cm^2^ (Table 1).

**Figure 6:**
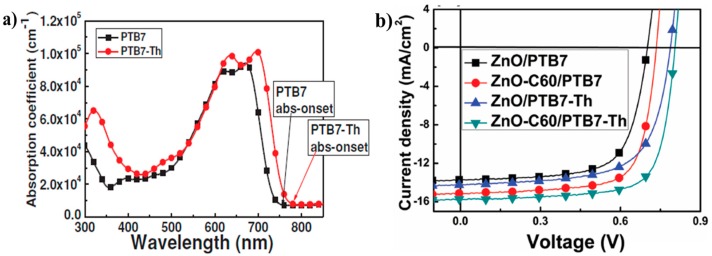
(**a**) The UV-visible absorption spectra of thin films of PTB7(P7) and PTB7-Th(P14); (**b**) *J*–*V* curves of the devices ITO/ZnO or ZnO-C_60_ (40 nm)/PTB7(P7):PC_71_BM or PTB7-Th(P14):PC_71_BM (1:1.5 *w/w*,100 nm)/MoO_3_ (10 nm)/Ag (100 nm). Reproduced with permission from Reference [53], Copyright 2013, Wiley.

The strategy by incorporating fluorine atom onto the thieno[3,4-*b*]thiophene unit to improve the coplanarity of the polymer main chain was developed by Liao et al. in 2103 [53]. To this end, polymer P14, which was always donated PTB7-Th, was synthesized. The UV-vis absorption spectrum revealed that P14 had the extended absorption by 25 nm compared to that of non-fluorinated P7 (Figure 6a). The photovoltaic cells were fabricated in an inverted construction with zinc oxide or fullerene derivative-doped zinc oxide as the cathode. The results showed that P14-based photovoltaic cells obtained a much better performance than that of P7 (Figure 6b). The best PCE of 9.35% with *V*_oc_ of 0.80 V, *J*_sc_ of 15.73 mA/cm^2^ and FF of 74.3% were achieved (Table 1), while a lower PCE of 8.21% for P7-based cells was found.

**Figure 7:**
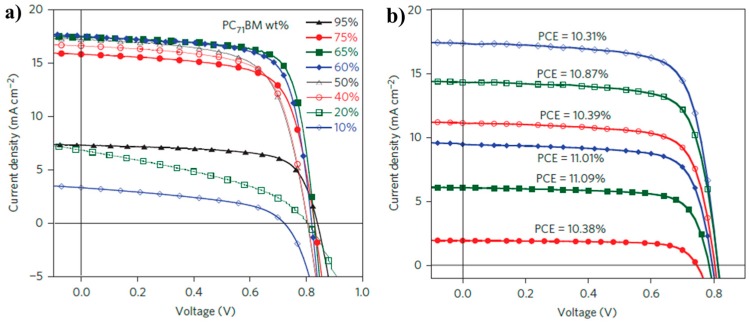
(**a**) The *J*–*V* characteristics of P14-based devices with different PC_71_BM weight fractions in the active layer; (**b**) *J*–*V* characteristics of a device with 65 wt % PC_71_BM in the active layer tested under different illumination conditions. Reproduced with permission from Reference [54], Copyright 2015, Nature publishing group.

In 2015, He et al. made another significant progress based on P14 via device engineering. They carefully investigated the effect of the weight fraction of PC_71_BM on device performance; they found that the *V*_oc_ increased to 0.841 from 0.750 V when the weight fraction of PC_71_BM increased to 90% form 10% (Figure 7a). With complicated device engineering, a best PCE of 10.28% was obtained with *V*_oc_ of 0.815 V, *J*_sc_ of 17.52 mA/cm^2^, FF of 72.01%. It was more interesting that this value would increase to 11.01% and 11.09% at lower light intensities of 501 and 316 W/m^2^, respectively (Figure 7b) [54].

Recently, Marks and coworkers proposed a new and highly region-selective direct C–H arylation polymerization (DARP) method, which eliminated the need for environmentally harmful, toxic organotin compounds. Using the DARP and Stille method, two kinds of P14 were synthesized. Interestingly, the result revealed that the two polymers showed similar chemical and physical properties. Under same device conditions, the polymers obtained the very similar PCEs of 8.19% and 8.24%, respectively. The results demonstrated that the DARP method may be an efficient and environmental method, which will become a new method for polymer synthesis in the future [55].

Using alkylthio chains to replace alkyl chains was a feasible strategy to root the PCE of solar cell by increasing the *J*_sc_ without sacrificing the high *V*_oc_. Developing the above thought, a polymer P15 was developed by Hou group. P15 had a strong absorption in the range from the wavelength of 500 to 800 nm. And the bandgap of 1.51 eV was calculated from the absorption onset of 820 nm. The CV result revealed the HOMO and LUMO levels of P15 were −5.33 and −3.5 eV, respectively. At the same condition, P15 revealed a slightly higher PCE of 9.48% with *V*_oc_ of 0.80 V, *J*_sc_ of 17.46 mA/cm^2^ and FF of 67.9% (Table 1), while a PCE of 7.42% was obtained for P14 [56].

Side chain engineering of BDT-based copolymers was also an effective way to improve photovoltaic performance of polymer donors. In 2016, Li and coworkers reported a new conjugated copolymer P16, which was composed of meta-alkoxyphenyl-substituted benzodithiophene and 2-ethylhexyl-3-fluorothieno[3,4-*b*]thiophene-2-carboxylate. The new polymer had many similar properties with P14. While being compared with P14, it displayed blue-shifted absorption spectra. And a deeper HOMO energy level of −5.45 eV was obtained for P16 via CV. In photovoltaic cells with PC_71_BM, a PCE of 9.0% was got with *V*_oc_ of 0.86 V, *J*_sc_ of 16.4 mA/cm^2^ and FF of 62.2% (Table 1). However, a PCE of 8.3% with *V*_oc_ of 0.78 V was obtained for P14 [57].

Besides the ester and ketone groups, the stronger electron-withdrawing group, sulfonyl, was also introduced onto the thienothiphene unit. The sulfonyl-substituted thienothiophene-based polymer, P17 (Figure 3), was reported by the Hou group [58]. P17 exhibited a broad absorption with a band-gap of 1.65 eV in thin film. The HOMO level was about −5.12 eV. The standard photovoltaic characterization showed a *V*_oc_ of 0.76 V, a *J*_sc_ of 14.1 mA/cm^2^ and a FF of 58%, with the overall PCE of 6.22% (Table 1). This result indicated that the sulfonyl group was a promising candidate for highly efficient polymer solar cell materials.

Molecular conformation of the polymer had a great influence on the morphology of the film made up of polymer, which affected the performance of solar cells. In 2013, Hou and coworkers used a DBD unit and thienothiphene unit with a sulfonyl group to synthesize a new polymer P18. DFT calculation showed P18 possessed a more linear conformation. At the same time, the distance between the adjacent side groups was big enough to allow the alkyls on the sulfonyl groups to stay in line with the backbone. The most important was that the intermolecular π–π stacking of the polymer will be enhanced so as to increase the FF. Finally, a PCE of 7.79% was obtained when blended with PC_71_BM (Table 1). Whereas, the solar cell based on the polymer with BDT unit as the donor only obtained a PCE of 5.93% [59].

In the same year, the Yu group reported two polymers P19 and P20 with DBD as a donor unit jointing fluorinated thieno[3,4-*b*]thiophene, which were different from the alkyl side chains hanged on the fluorinated thieno[3,4-*b*]thiophene unit. The two polymers both showed high molecules weights of 108.6 and 105.5 kDa. It was excited that P19 obtained a PCE of 7.6% with *V*_oc_ of 0.89V, *J*_sc_ of 13.0 mA/cm^2^ and FF of 65.3%. In contrast, P20 achieved a lower PCE of 4.9% with *V*_oc_ of 0.88 V, *J*_sc_ of 10.7 mA/cm^2^ and FF of 52.1% (Table 1), which was believed to be from the effect of the large alkyl chains in the fluorinated thieno[3,4-*b*]thiophene unit [60].

Beside benzodithiophene donor, Yu and coworkers also used the tetrathienoanthracene as the donor unit in the thieno[3,4-*b*]thiophene-based polymers [61]. With tuning the alkyl chains in tetrathienoanthracene unit, the polymer P21 with 2-butyloctyl side chain was found to have the highest PCE of 5.62% with a *J*_sc_ of 15.0 mA/cm^2^, a *V*_oc_ of 0.66 V and a FF of 58% blending with PC_61_BM (Table 1). When a longer alkyl chain was used, a poorer performance (2.98% in P22/PC_61_BM cell) was observed due to lower solubility compared with that branched alkyl chains. The grazing incidence wide-angle X-ray scattering (GIWAXS) studies found that the π-stacking of P21/PC_61_BM was in a face-down orientation and the π–π distance between P21 backbones was 3.6 Å, which was reduced by 0.5 Å when compared with P1–P7 polymer with same alkyl chains. The enhanced stacking was attributed to the extended π-conjugated monomer in P21. The enhanced stacking was one of the major reasons for the high efficiency of the P21/PC_61_BM cell.

Thiophene unit was normally employed in the polymer synthesis, in order to improve the charge carrier mobility. Polymer P23 with thiophene as spacer was developed by Hou and coworkers in 2012. The polymer possessed a bandgap of 1.59 eV with a much deeper HOMO energy level of −5.1 eV, owning to the introduction of strong electron-withdrawing fluorine atom. As expected, P23 showed a higher hole mobility of 2.76 × 10^−3^ than 4.56 × 10^−4^ cm^2^·V^−1^·s^−1^ of its counterpart polymer without thiophene spacer. Finally, the PCE of P23:PC_71_BM-based device revealed a PCE of 7.81% with a *V*_oc_ of 0.69 V, *J*_sc_ of 16.35 mA/cm^2^ and an enhanced FF of 66.3% (Table 1), while its counterpart polymer acquired a slightly low PCE of 6.11% with a low FF of 58% [62].

Hou’s group also incorporated fluorine atoms into BDT and thieno[3,4-*b*]thiophene units simultaneously, and synthesized a new polymer P24. The new polymer had an optical bandgap of 1.64 eV and possessed a slightly deeper HOMO energy level of −5.2 eV. To be congratulated, a high PCE of 8.6% with a *V*_oc_ of 0.78 V, a *J*_sc_ of 15.2 mA/cm^2^ and a FF of 72.3%, as PC_71_BM was used as the acceptor to fabricate solar cells (Table 1) [63].

### 2.2. Thieno[3,4-c]pyrrole-4,6-dione Based Polymers

**Figure 8:**
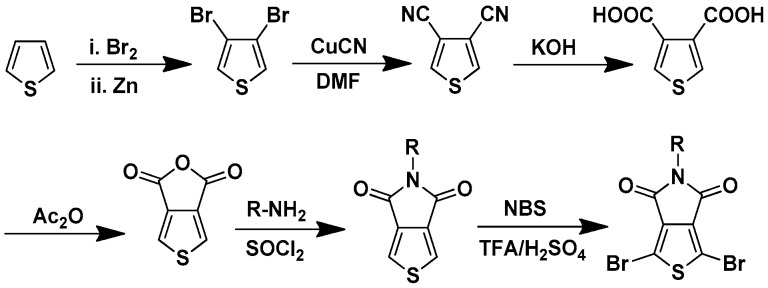
The synthetic route of bromide thieno[3,4-*c*]pyrrole-4,6-dione unit.

Recently, thieno[3,4-*c*]pyrrole-4,6-dione-based polymers have attracted much interest for highly efficient PSCs. The thieno[3,4-*c*]pyrrole-4,6-dione-based polymers were first synthesized by Tour and Zhang in 1997 [64]. Then, they mainly focused on the synthesis of low band-gap alternating polythiophenes [64,65]. Later, Pomerantz group and Bjørnholm group reported the homopolymers of thieno[3,4-*c*]pyrrole-4,6-dione with the studies of optical properties [66,67,68]. Combined with the X-ray diffraction (XRD) study, it was found that the homopolymers had more planar structures due to the efficient chains stacking. The thieno[3,4-*c*]pyrrole-4,6-dione was commonly synthesized via the straightforward steps starting from thiophene (Figure 8). The substitute (R) could be alkyl chain or aromatic ring, which both provided more rooms to tune the optical property and π-stacking of thieno[3,4-*b*]pyrrole-4,6-dione-based polymers [69].

**Figure 9:**
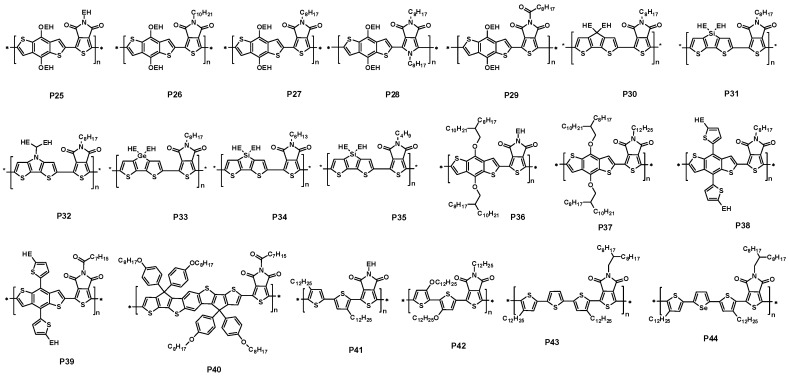
The chemical structures of the thieno[3,4-*c*]pyrrole-4,6-dione-based polymers for polymer solar cells (PSCs).

In 2010, the Leclerc group and the Jen group reported a thieno[3,4-*c*]pyrrole-4,6-dione-based polymer (P25, Figure 9) for PSCs at almost same time [70,71]. The polymer P25 contained an octyl-substituted thieno[3,4-*c*]pyrrole-4,6-dione unit as the acceptor and the 2-ethylhexyoxyl substituted benzodithiophene unit as the donor. The band-gap of P25 was found to be 1.82 eV, which was 0.2 eV lower than P3HT. The HOMO and LUMO energy levels of P25 estimated by cyclic voltammetry were −5.40 and −3.40 eV, respectively. The BHJ solar cells were fabricated in both conventional and inverted structures with the configuration of ITO/PEDOT:PSS/P25:PC_71_BM/LiF/Al and ITO/ZnO/C_70_-SAM/P25:PC_71_BM/ PEDOT:PSS/Ag, respectively. PCEs of 4.1%–5.5% in the conventional devices were reported with a *V*_oc_ of 0.85 V, a *J*_sc_ of 9.8 mA/cm^2^ and a FF of 50%–66% at the optimized P25:PC_71_BM weight ratio of 1:2 (Table 2). The PCE of inverted device with a C_70_ self-assemble monolayer was 4.2% with a *V*_oc_ of 0.87 V, a *J*_sc_ of 9.1 mA/cm^2^ and a FF of 54%.

**Figure 10:**
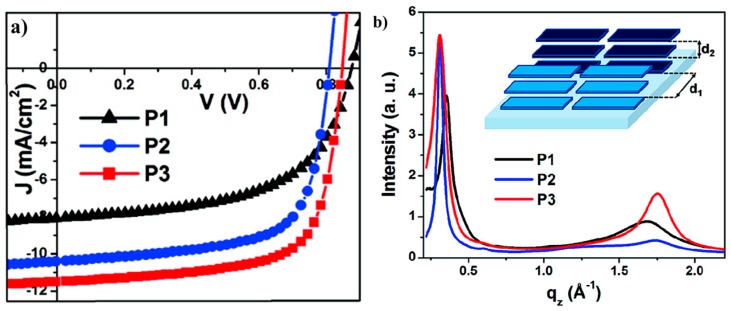
(**a**) Characteristic *J*–*V* curves of bulk heterojunction solar cells fabricated from P1(P25), P2(P26), and P3(P27) under illumination of AM 1.5 G, 100 mW/cm^2^; (**b**) out-of-plane linecuts of GIXS of P1(P25), P2(P26), and P3(P27) films. Inset: Schematic illustration of the face-on orientation of the polymers with the backbone parallel to the substrate. The lamellar spacing and the π-stacking distance are labeled *d*_1_ and *d*_2_, respectively. Reproduced with permission from Reference [72], Copyright 2010, American Chemical Society.

Later, P27 and other two polymers (P25 and P26, Figure 9) with different alkyl chains in thieno[3,4-*c*]pyrrole-4,6-dione unit were also reported by Fréchet and coworkers [72]. The lower band-gaps of 1.70–1.75 eV in these polymers were claimed. The initial photovoltaic tests of these polymers in the device structure of ITO/PEDOT:PSS/polymer:PC_61_BM/Ca/Al showed an interesting phenomenon. The P27 had the highest PCE of 6.3% with a *V*_oc_ of 0.86 V, a *J*_sc_ of 10.1 mA/cm^2^ and a FF of 68% (Table 2). However, with only a slight change on the side chain, the devices performance of P25/PC_61_BM and P26/PC_61_BM blend films showed a significant drop. Mreover, the PCEs of P25 and P26 devices were only 2.7% and 3.7% due to the significant loss on the *J*_sc_ and FF (Figure 10a). The low performance was attributed to the less polymer packing in the blend film. When replacing the ethylhexyl substituent on P25 with the dimethyloctyl and *n*-octyl analogues on P26 and P27, respectively, the π-stacking distances was reduced, and that was correlated well with increased device performance (Figure 10b). To further optimize the device performance, the mixture solvent of chorobenzene/DIO was used. It was interesting that the addition of DIO to the blend solution dramatically improved the devices performances of P25 and P26. The PCE was improved to 3.9% from 2.7% for P25 and 5.4% from 3.7% for P26 (Table 2). In contrast, the increment on P27 device was slight with the addition of DIO in the blend solution. The different behavior toward the addition of DIO was attributed to the different π–π stacking level of the blend film, which was mainly affected by the polymer side chains. The grazing incidence X-ray scattering (GIXS) studies found that the polymer backbones were oriented parallel to the substrates (so-called face-on orientation). By replacing the ethylhexyl substituent on P25 with the dimethyloctyl and *n*-octyl analogues on P26 and P27, respectively, the π-stacking distances were reduced from 3.8 to 3.6 Å. Further study on the blend film indicated that P27 had a more extended π-stacking than that P25 and P26. All these factors contributed to the high performance of P27-based devices.

Recently, Jeffries and co-workers used nitrogen atom to substitute the sulphur atom of TPD to synthesize PPD, which was a new acceptor unit. Therefore, a new polymer P28 was synthesized with PPD and BDT units. P28 has a wide bandgap of 2.20 eV, while PBDTTPD polymer showed the bandgap of 1.86 eV. Interestingly, P28 had the same HOMO energy level with PBDTTPD, which is about −5.50 eV. When fabricated, solar cells with PC_71_BM, P28 showed a lower PCE of 1.23% with *V*_oc_ of 0.76 V, *J*_sc_ of 3.9 mA/cm^2^, a FF of 42% (Table 2), whereas the PCE of 2.44% with *V*_oc_ of 0.69 V, *J*_sc_ of 7.8 mA/cm^2^ was achieved for PBDTTPD polymer. The low PCE was mainly attributed to the lower hole mobility in PBDTPPD polymer [73].

In 2013, a ketone component was introduced into TPD unit by Leclerc group, and a new polymer P29, which was copolymerized with TPD and BDT, was presented. This polymer acquired a moderate bandgap of 1.77 eV. Because of strong the electron-withdrawing unit, the new polymer possessed a deep HOMO energy level of −5.61 eV. A moderate PCE of 3.42% with a slightly high *V*_oc_ of 0.96 V, *J*_sc_ of 6.52 mA/cm^2^ and FF of 54% was achieved (Table 2), when fabricated an inverted bulk heterojunction configuration ITO/ZnO/polymer:PC_61_BM/MoO_3_/Ag [74].

The further developments of thieno[3,4-*c*]pyrrole-4,6-dione-based polymers were reported by Zhang et al. [75]. The polymers (P30–P32, Figure 9) with the C, Si, N-bridged dithiophene as the donor moiety were synthesized via Stille polycondensation. The band-gaps of P30 and P31 were 1.67 eV and 1.70 eV, respectively. The band-gap of P32 was further decreased to 1.59 eV due to the strong electron-donating property of pyrrole unit. The low-lying HOMO energy levels of −5.43 and −5.44 eV for P30 and P31 were observed. The HOMO energy level of P32 was −5.16 eV. The FET mobility of P30, P31 and P32 were reported to be 1.5 × 10^−3^, 6.0 × 10^−4^ and 3.9 × 10^−4^ cm^2^·V^−1^·s^−1^, respectively. The initial photovoltaic device processed from o-DCB solution showed a PCE of 3.74% for P30 with a *V*_oc_ of 0.80 V, a *J*_sc_ of 10.04 mA/cm^2^, and a FF of 47%. For P31 and P32 devices, the lower values of 1.18% and 0.91% were found, respectively. Although both devices showed a low performance, the *V*_oc_ of P31 and P32 devices were 0.90 and 0.71 V, respectively (Table 2). The high *V*_oc_ was due to the low-lying HOMO energy levels of the polymers. The device structure was ITO/PEDOT:PSS/polymer:PC_71_BM/Ca/Al. The optimized weight ratio of polymer to PC_71_BM was 1:2. The low performance was found to be related to the significant phase separation of P31/PC_71_BM and P32/PC_71_BM [75]. The large domain sizes increased the possibility of exciton recombination before reaching the interface of polymer and PC_71_BM. That was because of the limited diffusion length of exciton (10 nm), which could lead to the low photocurrent. The further optimization was processed by the addition of 1-chloronaphthalene into *o*-DCB solution, which played a negative role in the P30/PC_71_BM. However, the large enhancements in the P31/PC_71_BM and P32/PC_71_BM devices were found. The PCE of P31 device was improved to 2.13% from 1.18% with the increase on the *J*_sc_. Just like P31, the PSC also got an improvement PCE of 1.69% for P32. With the addition of 1-chloronaphthalene, the large domain sizes in the blend films of P31/PC_71_BM and P32/PC_71_BM disappeared, and the smooth features were found. The charge recombination, therefore, was reduced. As a result, the enhanced performances were found. In addition, Zhou et al. also reported an analogy of P32, in which a PCE of 1.65% was found [76].

**Figure 11:**
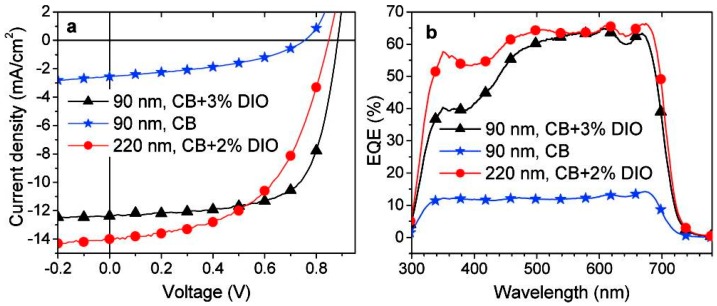
(**a**) The *J*-*V* curves of P31/PC_71_BM devices under different processing conditions; (**b**) EQE curves of the solar cells based on the 1:2 P31:PC_71_BM blend with different thicknesses fabricated from CB solutions (with no additive and 2 or 3 vol % DIO. Reproduced with permission from Reference [77], Copyright 2011, American Chemical Society.

Recently, a promising break on PCE of more than 7% using P31/PC71BM was reported by Hashimoto and coworkers [77]. The photovoltaic device structure was ITO/PEDOT:PSS/P31:PC71BM/BCP/Al. A thin layer (5 nm) of BCP between active layer and Al cathode was used as a hole/exciton blocking layer. When the device prepared from DCB/DIO (97/3, *v/v*) mixture solution, the best performance was observed with *V*_oc_ = 0.90 V, *J*_sc_ = 10.95 mA/cm^2^ and FF = 63%, resulting an overall PCE of 6.2%. The device performance was further improved by using chlorobenzene/DIO (97.3, *v/v*) mixture solution under same condition of DCB/DIO device. The best device exhibited a PCE of 7.3%, with a *V*_oc_ of 0.88 V, a *J*_sc_ of 12.2 mA/cm^2^ and a FF of 68% (Table 2). However, when the device prepared from chlorobenzene solution, a dramatic decrease on the performance was found. The PCE was less than 1% with the significant drop on *J*_sc_. The *J*–*V* curves of P31/PC_71_BM devices under different processing conditions were shown in Figure 11a. AFM studies revealed that there were large domains (up to 0.4 μm in diameter) in P31/PC_71_BM blend film prepared from the chlorobenzene solution. The large domains were not favorable for charge separation and transfer. After the addition of DIO, a much more uniform and finer domain structure with an average domain size of 20–40 nm was found in the blend film, so as to an improved device performance. It showed the importance of morphology control in the active layer of PSC once again. Further, the device based P31 and PC_71_BM, which had a thicker active layer of 220 nm with CB and DIO as additive and possessed an area of 1.0 cm^2^, was also reported and revealed a PCE of 6.1% with a *V*_oc_ of 0.85 V, a *J*_sc_ of 13.3 mA/cm^2^ and a FF of 54%. The enhanced *J*_sc_ was ascribed to the enhancement of EQE for P31 (Figure 11b). This promising result will make P31/PC_71_BM blend film be possible to fabricate PSCs by roll-to-roll printing techniques.

More recently, the dithienogermole-containing polymer (P33, Figure 9) copolymerizing with TPD unit was synthesized by Reynolds and coworkers [78]. With the Ge-substitute, P33 showed a red-shift in absorption compared with P31. The band-gap of P33 reached 1.69 eV. The HOMO energy level was also found to be −5.60 eV for P33, around 50 mV higher than that of P31. The inverted device with the structure of ITO/ZnO/P33:PC_71_BM/MoO_3_/Ag was fabricated. A poor performance was found in P33 device when the active layer was processed from chlorobenzene solution. However, adding 5% of DIO into chlorobenzene solution, a dramatic performance change was observed. With 5% of DIO, the PCE of P33 device reached as high as 7.3% in the inverted structure (Table 2). The significant performance enhancement was attributed to the morphology change with the addition of 5% of DIO in P33 solution. A small-scale phase separation on the order of the exciton diffusion length was revealed by TEM and AFM. Therefore, a higher *J*_sc_ and FF were observed as a result of the ideal morphology in P33/PC_71_BM when processing from CB/5% DIO solution. Through varying the side chains in the nitrogen atom of TPD, two new polymers P34 and P35 were prepared by Chu et al. Unfortunately, the two polymers possessed a slightly poor solubility for their shortened alkyl side chain hanged on the TPD, compared with P31. With the same device conformation, P31 showed a best PCE of 7.7% with *V*_oc_ of 0.91 V, a *J*_sc_ of 12.13 mA/cm^2^ and a FF of 70%. A slightly lower PCE of 5.3% and 6.4% were obtained for P34 and P35, respectively (Table 2) [79].

It was much more interesting that Zhang et al. also reported two theino[3,4-*c*]pyrrole-4,6-dione and benzodithiophene-based polymers (P36 and P37, Figure 9), which were with a more bulk and longer branched alkyl chains in the benzodithiophene unit [80]. A similar band-gap (1.84 eV) was achieved in both polymers. The HOMO energy levels of −5.42 and −5.44 eV for P36 and P37 were observed with the LUMO energy levels of −3.60 and −3.58 eV, respectively. The photovoltaic performance was characterized with the structures of ITO/PEDOT:PSS/polymer:PC_71_BM (1:2, *w/w*)/LiF/Al. It was interesting that both P36 and P37 devices exhibited a high *V*_oc_ of 0.97 and 0.91 V, respectively. The low efficiency mainly derived from the low *J*_sc_, which may be due to the unfeasible morphology. After the addition of DIO (2% by volume) to P36/PC_71_BM and P37/PC_71_BM blend solution, the large enhancement on device performances was found. The P36-based device showed an improved PCE of 3.42% with a *V*_oc_ of 0.93 V, a *J*_sc_ of 6.58 mA/cm^2^ and a FF of 56%. The highest PCE was found to be 4.79% with a *V*_oc_ of 0.91 V, a *J*_sc_ of 10.34 mA/cm^2^ and a FF of 51% in P37-based device (Table 2). The more feasible morphologies of P36/PC_71_BM and P37/PC_71_BM blend films with the addition of DIO caused the enhancement on *J*_sc_, and further leaded to the improved performance. It was noted that the alkyl chains may give an effect on the *V*_oc_, even the similar HOMO levels were observed.

It is well known that, when an alkylthienyl side chain was used to replace alkyl side chain in BDT unit, the solar cells based in the polymer always obtained a high *V*_oc_ and a better PCE in the same device fabrication. To this end, Ma group presented a new polymer P38 in which alkylthienyl side chain was introduced on BDT unit in 2013. As expected, a slightly deep HOMO level of −5.61 eV was got. And when fabricated solar cell with PC_61_BM as the acceptor, the device exhibited a moderate PCE of 6.17% with a *V*_oc_ of 1.00 V, a *J*_sc_ of 9.79 mA/cm^2^ and a FF of 63% (Table 2) [81].

**Figure 12:**
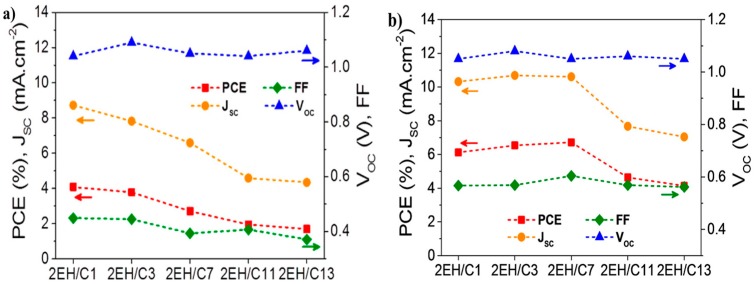
The *J*_sc_, *V*_oc_, FF and PCE for optimized BHJ solar cells fabricated from the PBD(T)TPD(CO)derivatives-(2EH/C1), (2EH/C3), (2EH/C7)(P39), (2EH/C11), and (2EH/C13)-under AM1.5G: (**a**) Devices cast from CF, no CN additive; (**b**) with 3% CN additive (*v/v*) Reproduced with permission from Reference [82], Copyright 2014, American Chemical Society.

A ketone group was also introduced to modify TPD with the aim of increasing the *V*_oc_. Beaujuge and co-workers used thienyl BDT with *N*-alkyloyl-substituted TPD unit to produce a series of polymers. These polymers have the different alkyloyl side chains in TPD unit. Among these polymers, P39 showed a PCE of 2.7% without additive (Figure 12a). In contrast, a better PCE of 6.7% with an outstanding high *V*_oc_ of 1.05 V, *J*_sc_ of 10.6 mA/cm^2^ and a FF of 60% was achieved (Table 2) when P39 was fabricated on solar cells with PC_71_BM and with 3% CN additive (Figure 12b) [82].

To extend the conjugation length of the polymer, Chen et al. developed the heptacyclic benzodi(cyclopentadithiophene) as the donor unit by covalently rigidifying the central BDT subunit with two external thiophenes, which was then used as the donor unit to synthesize a new polymer P40. As expected, the polymer presented a rigid plane, which will benefit for the charge carrier transport. When solar cells were fabricated with PC_71_BM and dimethylsulfoxide as solvent additive, P40 showed a PCE of 6.6% (Table 2). In contrast, the device based the corresponding non-fused polymer analogue shows a much lower PCE of 0.2% [83].

In addition to benzodithiophene as donor, various other donors were also incorporated into the thieno[3,4-*c*]pyrrole-4,6-dione-based polymers. For example, Wei and coworkers reported the high crystallized polymer P41 (Figure 9), which contained a symmetrical electron-donating bi(dodecyl)thiophene and a 2-ethylhexyl-substituted thieno[3,4-*c*]pyrrole-4,6-dione [84]. P41 showed a crystallization point at 268 °C and a melting point at 297 °C. The face-on π–π stacking distance of P41 was 3.6 Å. The absorption maximum of P41 in film state showed a large red-shift (104 nm) compared with that in chloroform solution. The band-gap in film state was found to be 1.82 eV. The HOMO and LUMO energy levels of P41 were −5.56 and −3.10 eV, respectively. A lower-lying HOMO level was found in the comparison with that of P25–P27. The photovoltaic cell in the configuration of ITO/PEDOT:PSS/P10:PC_61_BM/Al was fabricated. The optimized device exhibited a *V*_oc_ of 0.95 V, a *J*_sc_ of 8.02 mA/cm^2^ and a FF of 62%. Eventually, a moderate PCE of 4.7% was got for P41 (Table 2). This high *V*_oc_ was benefiting from the low-lying HOMO energy level. It was noted that the device performance came from Al cathode and P41/PC_61_BM blend. A higher performance will be highly expected if a low work-function metal (i.e., Ca) and PC_71_BM were used. Watson and coworkers also reported a similar polymer P42 (Figure 9), which contained the alkyloxy side chains on the bithiophene unit [85]. Because of the strong electron-donating property of alkyloxyl side chain, the band-gap of P42 was reduced to 1.50, 0.32 eV lower than that of P41. The HOMO and LUMO energy levels also increased to −4.85 and −3.35 eV, respectively. The FET mobility of P42 was reported to be 8.3 × 10^−4^ cm^2^·V^−1^·s^−1^ after annealing at 200 °C for 10 min. The photovoltaic device at the structure of ITO/PEDOT:PSS/P42:PC_71_BM/LiF/Al showed a PCE of 1.44% with a *V*_oc_ of 0.41 V, a *J*_sc_ of 7.39 mA/cm^2^ and a FF of 48% (Table 2). The low *V*_oc_ was believed to be ascribed to the high-lying HOMO level of P42.

In 2012, the Heeger group presented a new polymer P43 based on terthiophene-thienopyrrolodione alternating building blocks. When blended with PC_70_BM, the solar cell achieved a PCE of 5.77 (Table 2) [86]. In the same year, the Heeger group replaced thiophene with selenophene in a TPD unit to synthesize a new polymer P44 again. Compared with P43, P44 had a slightly high HOMO energy level of −5.49 eV. When Clevious PVPAI4083 was used as the hole-transport interlayer , the solar cell based P44:PC_71_BM as the active layer obtained a PCE of 5.80% with a *V*_oc_ of 0.88 V, a *J*_sc_ of 10.74 mA/cm^2^ and a FF of 62% (Table 2) [87].

**Figure 13:**
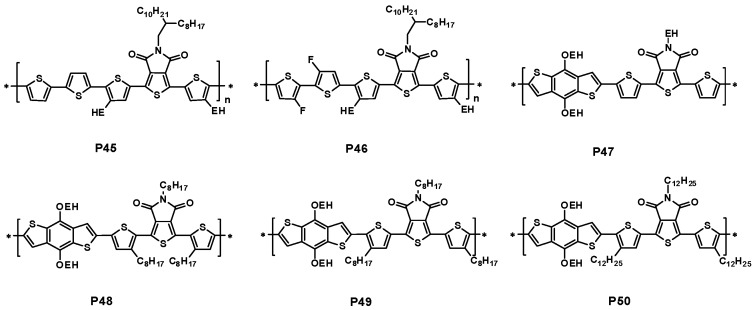
The chemical structures of the thieno[3,4-*c*]pyrrole-4,6-dione-based D-π-A polymers for PSCs.

**Figure 14:**
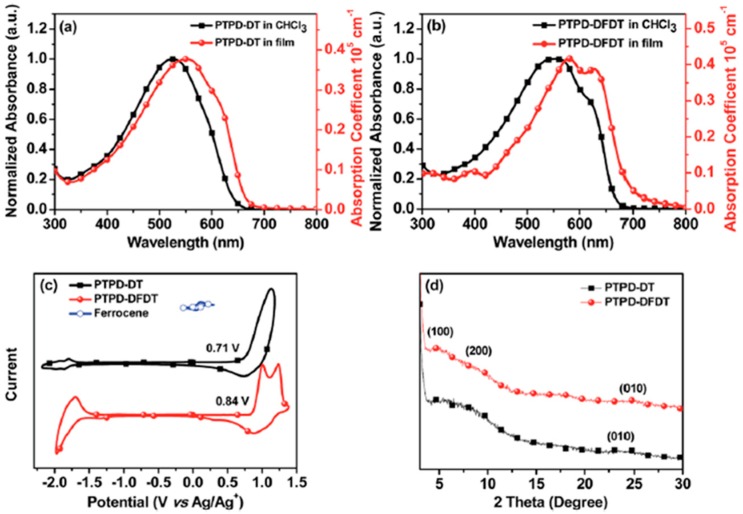
UV-vis absorption spectra of PTPD-DT(P45) (**a**) and PTPD-DFDT(P46) (**b**) in *o*-DCB solution and solid film; (**c**) cyclic voltammograms of PTPD-DT(P45) and PTPD-DFDT(P46) films on a platinum electrode measured in 0.1 mol·L^−^^1^ Bu_4_NPF_6_ acetonitrile solution at a scan rate of 50 mV·s^−^^1^. (**d**) X-ray diffraction patterns of the polymer films cast from *o*-DCB onto Si substrates. Reproduced with permission from Reference [88], Copyright 2016, Royal Society of Chemistry.

As discussed above, introducing a fluorine atom onto the polymer backbone has been a common strategy to increase *V*_oc_ of solar cell. Recently, Li and coworkers reported two TPD-based polythiophene derivative polymers with the fluorine substitutes on bithiophene donor (P46). Compared with P45, P46 (Figure 13) revealed a slightly lower HOMO energy level of −5.55 eV and a much broader absorption in the wavelength range from 300 to 700 nm (Figure 14). Furthermore, the XRD studies revealed that P46 showed a more compact π–π stacking than P45, which should be ascribed to the higher coplanarity and stronger interchain interaction of P46 benefitting from fluorine substitution. When blended with PC_71_BM, a PCE of 5.52% along with an encouraging *V*_oc_ of 0.96 V was obtained for P46, while a PCE of 2.32% was obtained for P45 with a *J*_sc_ of 5.72 mA/cm^2^ (Table 3) [88].

The Leclerc group and the Zhang group synthesized a series of new polymers with a thiophene spacer on each side of thieno[3,4-*c*]pyrrole-4,6-dione unit, then connecting with the 2-ethylhexyoxyl-substituted benzodithiophene (P47–P50, Figure 13) [89,90]. The polymer P47 was insoluble in most organic solvents, partially due to the stronger molecular packing. Further optimizations on solubility through introducing the alkyl chains onto thiophene unit were reported. There were two positions in the thiophene unit available for the modification. P48 and P49 were the two polymers with same alkyl substitutes, but with a different position in the thiophene unit. P48 and P49 had a similar band-gap of 1.83 eV. The HOMO energy levels of P48 and P49 were determined to be −5.56 and −5.66 eV, respectively. The photovoltaic cell containing P48/PC_61_BM blend film under the device structure of ITO/PEDOT:PSS/P48:PC_61_BM/Al showed a PCE of 3.9%, a *V*_oc_ of 0.89 V, a *J*_sc_ of 7.6 mA/cm^2^ and a FF of 57% (Table 3). However, the device from P49/PC_61_BM blend under the same device structure showed a dramatic decrease in performance with a PCE of only 0.2% (Table 3). It was noted that both devices of P48 and P49 used Al as the cathode. In another report, P50 with a larger alkyl chain possessed a similar bandgap as P49. The reported HOMO energy level of P50 was −5.35 eV. The initial optimized PCE of P49/PC_71_BM device was 2.05% in the device structure of ITO/PEDOT:PSS/P50:PC_71_BM/LiF/Al. Further optimization by the addition of DIO showed a PCE of 2.68% with a slight decreased *V*_oc_ of 0.83 V, an increased *J*_sc_ of 5.28 mA/cm^2^ and an improved FF of 61% (Table 3). The performances of P47–P50-based devices were far lower than that of P25 and P27-based devices, more understandings on the structure-performance relationship are highly necessary for these polymers.

### 2.3. Polymers Containing 2,1,3-Benzothiadiazole

In donor-acceptor alternating polymers for PSCs, 2,1,3-benzothiadiazole is one of the most investigated and widely used acceptors. In the past decade, many polymers based on 2,1,3-benzothiadiazole with the donor unit such as fluorene [91,92], carbazole [93,94,95], indolocarbazole [96,97], silafluorene[98,99], cyclopentadithiophene [26,100,101,102,103], dithienosilole [104,105], dithienopyrole [106] and so on have been reported. Some of them showed promising photovoltaic performance with the various devices engineering. For example, carbazole units were proved to be an excellent donor in a class of polymers with PCEs exceeding 6% [107]. In 2009, the Heeger group reported a new polymer P51 based carbazole and di-thienyl—benzothiadiazole units. This polymer revealed a moderate PCE of 6.1% (Table 4) when fabricated solar cell devices with PC_71_BM as the acceptor and TiO_x_ layer as an optical spacer. Most important, the P51:PC_71_BM-based solar cell showed an outstanding internal quantum efficiency about 90% among the entire absorption spectrum, making it clear that almost all excitons were separated to form pair of charge carriers and that all the charge carriers were collected at the electrodes [108].

Thiophene was a common and accessible unit, which was always used to synthesize polymers with other acceptors. A new polymer P52 based quaterthiophen and benzothiadiazole units were developed by Ong et al. The bandgap of P52 was about 1.59 eV, and HOMO energy level of −5.18 eV. When fabricated solar with PC_71_BM, the devices demonstrated high PCEs between 5.83% and 6.26% (Table 4) [109].

Indacenodithiophene (IDT) has been paid much attention in the design of new conjugated copolymers for the application in the solar cells [110]. The coplanarity of the IDT unit could enhance the interchain interaction of the polymers and be expected to have higher hole mobility [111]. A polymer P53 composed of BT jointing with alkyl-substituted indacenodithiophene was presented by Zhang et al. P53 revealed a better solubility in many organic solvents. As expected, a higher hole mobility of 2.24 × 10^−3^ cm^2^·V^−1^·s^−1^ was obtained by the space charge limit current (SCLC) method using a device structure of ITO/PEDOT:PSS/polymer/Au. Eventually, the PCE of solar cell made up of P53:PC_71_BM blend showed a PCE of 6.17% with a *J*_sc_ of 13.27 mA/cm^2^, a *V*_oc_ of 0.82 V, and a FF of 56.9% (Table 4) [112].

It is reasonable to believe that incorporating larger polycyclic aromatics into the conjugated polymers should be beneficial to increasing the charge carrier transport properties [113]. In 2016, Huang et al. used thieno[3,2-*b*]indole (TI) unit 2,1,3-benzodiathiazole (BT) unit to synthesize two conjugated polymers P54 and P55. Different from P54, P55 possessed a thiophene as the spacer. The author found the thiophene spacer had a dramatic influence on the physical and electrochemical properties of P55, when compared with P54. The two polymers both showed strong absorption from 300 to 750 nm in the UV-vis spectra. While P55 exhibited somewhat more red-shift compared with P54. So a slightly low bandgap of 1.60 eV for P55 than the one of 1.66 eV for P54 was obtained. The HOMO energy levels were −5.27 and −5.22 eV for P54 and P55, respectively. When solar cells were fabricated on the structure of ITO/PEDOT:PSS/polymer:PC_71_BM/Ca/Al, P55 acquired a higher PCE of 5.83% with *J*_sc_ of 13.92 mA/cm^2^, a *V*_oc_ of 0.69 V, and a FF of 61.8%.For P54, a PCE of 1.61% was achieved due to the lower *J*_sc_ of 5.86 mA/cm^2^ (Table 4) [114].

**Figure 15:**
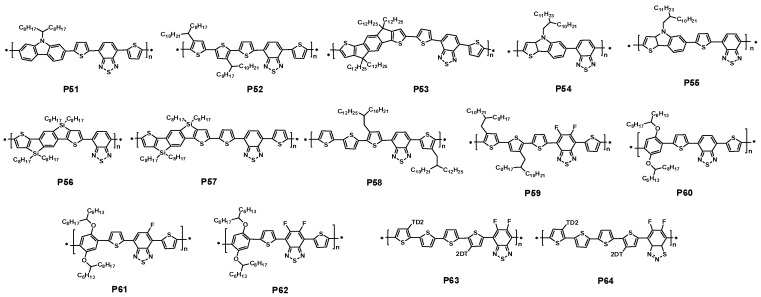
The chemical structures of 2,1,3-benzothiadiazole-based polymers for PSCs.

When the carbon atom was replaced by a silicon atom in the IDT unit, two polymers P56 and P57 (Figure 15) were reported by Jen and coworkers [115]. It was noted that P56 was also synthesized by McCulloch’s group at almost the same time [116]. The silaindacenodithiophene donor was synthesized via multi-steps from 2-bromothiophene and 1,4-dibromo-2,5-diiodobenzene. The band-gaps of P56 and P57 were 1.80 and 1.70 eV, respectively, which were slightly larger than their counterpart polymers. The HOMO and LUMO energy levels of P56 and P57 were −5.32 and −3.25 eV; −5.25 and −3.55 eV, respectively. The HOMO energy levels were similar to their counterpart of indacenodithiophene-based polymers. The two polymers both showed a high FET mobility of up to 8 × 10^−3^ and 1.2 × 10^−2^ cm^2^·V^−1^·s^−1^ for P56 and P57, respectively. The photovoltaic properties of P56 and P57 were studied in PSCs with a device structure of ITO/PEDOT:PSS/polymer:PC_71_BM/Ca/Al. The resulting PCE for P56 was 3.49% with a *V*_oc_ of 0.81 V, a *J*_sc_ of 8.86 mA/cm^2^ and a FF of 48.8% (Table 4) [115]. In the report of McCulloch group, the device with the same configuration gave a *V*_oc_ of 0.88V, a *J*_sc_ of 9.39 mA/cm^2^ and a FF of 52%, resulting in a PCE of 4.3% (Table 4) [116]. In the case of P57, a similar photovoltaic performance was achieved. The PCE of the P57/PC_71_BM blend system is 3.64%, showing a *V*_oc_ of 0.80 V, a *J*_sc_ of 8.8 mA/cm^2^ and a FF of 51.6%. It was strange that these Si-analogous polymers showed lower performance compared with the C-analogous polymers.

**Figure 16:**
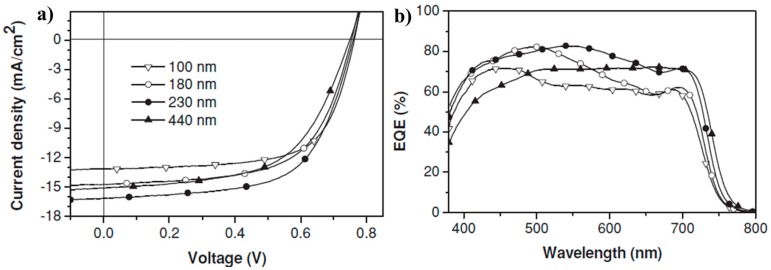
*J*–*V* characteristics (**a**) and EQE curves (**b**) of P58 in the inverted solar cells with active layer thickness range from 100 to 440 nm. Reproduced with permission from Reference [117], Copyright 2014, Wiley.

In 2014, a polymer P58 based 5,6-difluorobenzothiadiazole unit and quarterthiophene with solubilizing alkyl chains attached on the two terminal thiophene rings was reported by the Cao group. A high hole mobility of 1.92 cm^2^·V^−1^·s^−1^ in OFET was obtained, owning to the strong interchain aggregation of P58. When solar cells were fabricated with PC_71_BM, the effect of the thickness of the active layer on the photovoltaic performance was also studied. Finally, a best PCE of 7.64% was obtained with a *J*_sc_ of 16.2 mA/cm^2^, a *V*_oc_ of 0.76 V, and a FF of 62.1% (Table 4) with 230 nm active layer (Figure 16a), which was ascribed to the enhanced EQE (Figure 16b) [117]. The Hsu group used the same backbone of the polymer P58, but introduced fluorine atom into the benzothiadiazole unit to synthesize a polymer P59. Owning to long-range ordered lamellar structure and better p–p stacking, P59 possessed a high hole mobility of 0.29 cm^2^·V^−1^·s^−1^. Finally, when solar cells were fabricated in the conventional and inverted form, PCEs of 6.41% and 6.82% were obtained respectively (Table 4) [118].

Three semi-crystalline and low bandgap (LBG) polymers P60, P61 and P62 based on dialkoxyphenylene and BT-based unit, were developed by Nguyen et al. The three polymers all had a similar bandgap. Whereas they possessed a slightly different HOMO energy levels, which were −5.29, −5.35 and −5.45 eV. When fabricated solar cells, a best PCE of 7.26% was obtained for P62 with a *J*_sc_ of 11.40 mA/cm^2^, a *V*_oc_ of 0.86 V, and a FF of 74%. On the other hand, a PCE of 5.08% and 5.11% were got for P60 and P61, respectively (Table 4) [119].

Recently, 5,6-difluoro-benzo[d][2,1,3]thiadiazole (dfBT) and its isomer, 5,6-difluoro-benzo [d][1,2,3]thiadiazole (isoBT) were also introduced to synthesize P63 and P64 with tetrathiophene donor by Facchetti and coworkers. In comparison with P63, the absorption of P64 shows a blue shift about 70 nm. Meanwhile, P64 obtained a slightly deeper HOMO energy level of −5.7 eV than P63. It was very encouraging that the photovoltaic cell based on P64 showed a PCE of 9.0% in the structure ITO/PEDOT/Polymer:PC_71_BM/LiF/Al, whereas P63 obtained a PCE of 8.8% (Table 4) [120].

**Figure 17:**
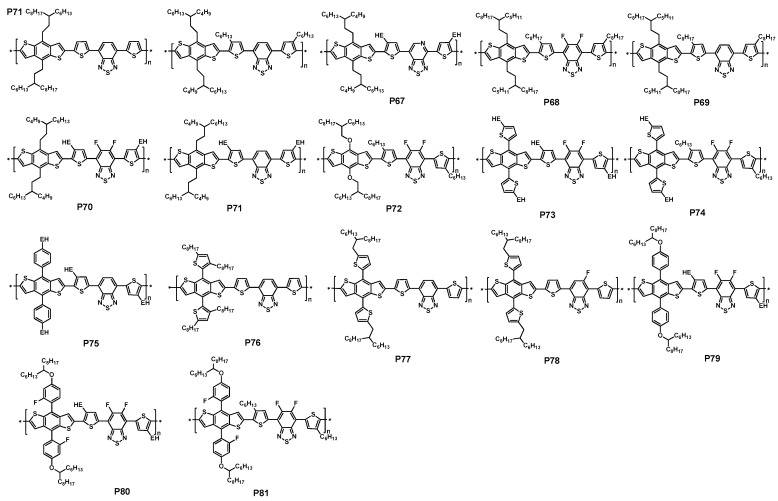
The chemical structures of BDT-π-2,1,3-benzothiadiazole-based D-π-A polymers for PSCs.

You et al. synthesized two polymers (P65 and P66, Figure 17) with the different on side chains on the BDT unit [121]. The band-gaps of P65 and P66 were alike about 1.7 eV. It was interesting that P65 showed a more pronounced shoulder at approximately 650 nm while there was no such result for P66, which meant a slightly increased π-stacking and the extension of the conjugation in the thin film of P65. The HOMO and LUMO energy levels of −5.33 and −3.17 eV were reported for P65. After addition of the electron-donating hexyl chain in thiophene unit, the HOMO and LUMO energy levels of P66 were increased to −5.26 and −2.96 eV, respectively. The preliminary photovoltaic tests in P65/PC_61_BM (1:1, *w/w*) blend showed a PCE of 3.85% with a *V*_oc_ of 0.83 V, a *J*_sc_ of 7.70 mA/cm^2^ and a FF of 59.6%. In the case of P66, the PCE was increased to 4.31%, showing a *V*_oc_ of 0.81 V, a *J*_sc_ of 9.70 mA/cm^2^ and a FF of 54.8% (Table 5). The increased performance in P66 was partially attributed to be a high molecular weight (P65:*M*_n_ = 5.6 kDa, P66:*M*_n_ = 21.9 kDa) and have good solubility of P66 over P65. There was no P65 and P66/PC_71_BM-based device performance reported, which could be used for comparison with P59-based device to illustrate the effect of side chains on the photovoltaic property.

Thiadiazolo[3,4-*c*]pyridine, an analogous compound of the benzothiadiazole, was also incorporated with these benzodithiophene units by Zhou et al. [122]. Thiadiazolo[3,4-*c*]pyridine was a stronger acceptor than benzothiadiazole because of the p-electron deficient pyridine unit; therefore, a lower band-gap of the resulting polymers would be expected. To this end, polymers P67 containing thiadiazolo[3,4-*c*]pyridine was synthesized from the corresponding monomer (Figure 17). The low band-gap of P67 was calculated to be −1.51 eV from the absorption onset of film, which was 0.2 eV lower than the corresponding analogous benzothiadiazole-based polymer. The HOMO energy level P67 was −5.47 eV. The photovoltaic property of P67 was studied under the device structure of ITO/PEDOT:PSS/polymer:PC_61_BM/Ca/Al. It was encouraging that the P67 showed a good performance with a *V*_oc_ of 0.85 V, a *J*_sc_ of 12.78 mA/cm^2^ and a FF of 58.2%, which resulted in a moderate PCE of 6.32% (Table 5).

As it had been demonstrated by Yu and coworkers [44] that the introduction of fluorine atom was an effective way to enhance the *V*_oc_ of BHJ cell in the thienothiophene polymers. Recently, this method had also been applied onto benzothiadiazole unit, in which the hydrogen atoms in the 4 and 5 positions of benzothiadiazole unit was replaced with the fluorine atom, by Li et al., Zhou et al. and Zhang et al. [123,124,125]. The fluorine-substituted benzothiadiazole was synthesized via several steps from 1,4-dibromo-2-fluorobenzene or 1,2-difluorobenzene. The corresponding F-substituted benzothiadiazole-based polymers P68, P70 were synthesized through Stille polymerization (Figure 17). It was noted that P68 and its non-fluorinated analogous P69 were synthesized by You et al. P70 and its non-fluorinated analogous P71 were reported by Li et al. [123]. In the case of P68, the UV-vis absorption in thin film showed 50 nm blue-shift compared with that of P69. The optical band-gap calculated from the absorption onset in thin film was therefore reduced to 1.70 eV, which was slightly larger than that of P69 [124]. In P70 and P71, however, a low optical band-gap of 1.60 eV was reported [123]. It was unclear how the absorption onset was taken in both papers, so it was difficult to illustrate the possible side chain or polymer chain stacking effect on the polymer’s band-gap. The HOMO and LUMO energy levels of P68 and P69 were −5.54 and −3.33 eV; −5.40 and −3.13 eV, respectively. Both HOMO and LUMO levels were lowered after the introduction of fluorine atoms in benzodiathiazole unit. Similarly, the HOMO energy level of P70 was reduced to −5.48 from −5.30 eV in P71. The BHJ devices of P68 and P69 were fabricated in the configuration of ITO/PEDOT:PSS/P68 or P69:PC_61_BM (1:1, *w/w*)/Ca/Al. In the P68/PC_61_BM blend system, a promising and high PCE of up to 7.2% was achieved with a *V*_oc_ of 0.91 V, a *J*_sc_ of 12.91 mA/cm^2^ and a FF of 61.2% [124]. The thickness of active layer was 190 nm, which was in the similar range of the optimized P3HT/PC_61_BM device and but almost double of the typical PSCs. The P69/PC_61_BM device with a thickness of 170 nm showed a maximum PCE of 5.0%, a *V*_oc_ of 0.87 V, a *J*_sc_ of 10.03 mA/cm^2^ and a FF of 57.3% [124]. It can be seen that all parameters were increased. The increased *V*_oc_ in P68 was contributed to by the lower-lying HOMO energy level. The increased *J*_sc_ was attributed to the higher absorption coefficient in P68/PC_61_BM blend film than that in P69/PC_61_BM blend. The increased FF was believed to come from the balanced charge transport and the improved morphology of P68/PC_61_BM blend system. In the case of P70, the device structure of ITO/PEDOT:PSS/P70:PC_71_BM/LiF/Al, which was different with that of P68 and P69, was used to investigated the photovoltaic property [123]. The P70/PC_71_BM-based device exhibited a PCE of 3.40% with a *V*_oc_ of 0.69 V, a *J*_sc_ of 8.89 mA/cm^2^ and a FF of 55.4%. It was noted that the active layer was processed from the 1,2,4-trichlorobenzene solution of P70/PC_71_BM due to the limited solubility of P70. The optimized P71-based device only gave a PCE of 1.88% with the low *J*_sc_ and FF, but having a same *V*_oc_ compared to that of P32 [123]. It is known that the processing condition may also affect the *V*_oc_, so that it was difficult to compare this in both devices of P70 and its non-fluorinated analogous polymer P71.

The Chen group reported three polymers P72, P73 and P74 based fluorinated benzothiadiazole with BDT hanged different side chains. The three polymers possessed similar bandgaps of 1.70, 1.76 and 1.72 eV as the almost same as the HOMO energy level of −5.31, −5.34 and −5.33 eV. The solar cell fabricated with PC_71_BM, P72 obtained a slightly higher *J*_sc_ of 15.38 mA/cm^2^, which was attributed to the strong EQE in the wavelength range from 300 to 750 nm. In contrast, a slightly lower *J*_sc_ of 13.17 and 11.87 mA/cm^2^ was obtained for P73 and P74, respectively. Eventually, P72 obtained a satisfactory PCE of 8.30% with a *V*_oc_ of 0.78 V, and a FF of 69.2%, while a PCE of 6.2% and 4.46% was obtained for P73 and P74, respectively (Table 5) [126].

Alkylphenyl substituted BDT (BDTP) has attracted much attention as a weak electron-donating unit with large π-conjugated area and good planarity. In 2013, the Hou group reported a polymer P75 based BT with alkylphenyl substituted BDT. An optical bandgap of 1.70 eV was obtained for P75. A deep HOMO level of −5.35 eV was meanwhile obtained. The hole mobility of the copolymer was measured by space charge limit current (SCLC) method by using a device structure of ITO/PEDOT:PSS/polymer/Au. Finally, a high hole mobility of 8.89 × 10^−2^ cm^2^·V^−1^·s^−1^ was obtained. When solar devices were fabricated with PC_71_BM, a PCE of 8.07% with a *V*_OC_ of 0.88 V, a *J*_SC_ of 12.94 mAcm^−2^ and a FF of 70.9%, was obtained (Table 5) [127].

Even it has been widely shown that benzodithiophene was a promising donor unit for efficient PSCs, unfortunately, low efficiencies were occasionally found in the poly(benzodithiophene-*alt*-benzothiadiazole)s in some examples. To alleviate this dilemma, Yang and coworkers reported a copolymer of poly[4,8-bis(2,5-dioctyl-2-thienyl)-benzo[1,2-b:4,5-b′]dithiophene-*alt*-[4,7-bis(2-thienyl)-2,1,3-benzothiadiazole)-5,5′-diyl]] (P76, Figure 17), which contained a thiophene spacer between benzodithiophene and benzothiadiazole units [105]. P76 showed a band-gap of 1.75 eV in the film state. The HOMO and LUMO energy levels of P76 were −5.31 and −3.44 eV, respectively. The photovoltaic cell was fabricated under the configuration of ITO/PEDOT:PSS/P76:PC_71_BM/Ca/Al. Device measurement obtained a promising PCE of up to 5.66% with a high *V*_oc_ of 0.92 V, a *J*_sc_ of 10.7 mA/cm^2^ and a FF of 57.5% (Table 5). This was one of the highest values reported at that time. Continuing to keep the whole backbone of polymer of P76, the Jo group incorporated fluorine atoms into the benzothiadiazole unit, and synthesized a new polymers P78 [128]. P77 was also synthesized with the same structure of P78, but there was not fluorine atom on the benzothiadiazole unit for P77. The two polymers possessed the similar bandgaps, which were 1.78 and 1.80 eV, respectively. Compared with P77, P78 had a deeper HOMO energy level of −5.48 eV, owning to the introduction of fluorine atom. When fabricated solar cells with PC_71_BM, P78 obtained a higher PCE of 7.27% with a *V*_oc_ of 0.95 V, a *J*_sc_ of 11.95 mA/cm^2^ and a FF of 64%. As the counterpart, P77 got a slightly low PCE of 6.92% with a slightly lower *V*_oc_ of 0.92 V, a *J*_sc_ of 11.61 mA/cm^2^, but obtained an increased FF of 65% (Table 5).

**Figure 18:**
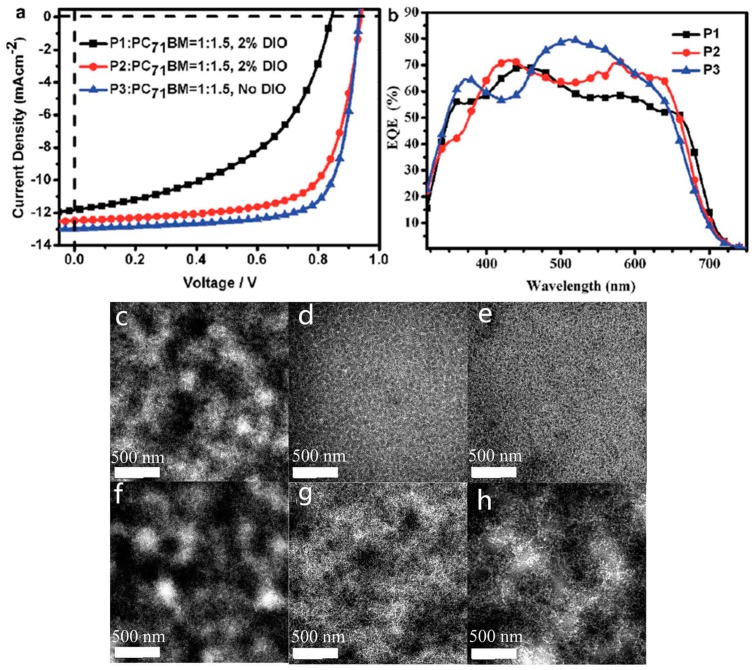
(**a**) Optimized *J*–V curves of PSCs based on the polymer/PC_71_BM blend film; (**b**) EQE spectra of the optimized devices. TEM images of the active layers of P1(P79)/PC_71_BM (**c**); P1(P79)/PC_71_BM with 2% DIO (**d**); P2(P8)0/PC_71_BM (**e**); P2(P80)/PC_71_BM with 2% DIO (**f**); P3(P81)/PC_71_BM (**g**); P3(P81)/PC_71_BM with 2% DIO (**h**). Reproduced with permission from Reference [129], Copyright 2016, Royal Society of Chemistry.

Recently, the Yang group used *p*-alkoxyphenyl-substitutedbenzo[1,2-b:4,5-b0]dithiophene (BDT) units, with and without fluorine, and 5,6-difluoro-4,7-di(4-(2-ethylhexyl)-2-thienyl)-2,1,3-benzothiadiazole to construct three polymers P79, P80 and P81. These polymers possessed a bandgap of 1.75, 1.82 and 1.82 eV, respectively. The result of CV revealed the HOMO energy of these three polymers was −5.5, −5.63 and −5.56 eV, respectively. When solar cells were fabricated, P81 possessed a higher *J*_sc_ of 12.97 mA/cm^2^, which was because of the enhancement of EQE (Figure 18b). At the same time, because of the highest hole mobility in the three polymers, a highest FF of 74.49% was also obtained for P81. All these factors contributed to a best PCE of 9.02% among three polymers for P81, while the PCEs of 4.91% and 8.1% were obtained for P79 and P80, respectively (Table 5) (Figure 18a). As shown in Figure 18, the blend film of P79 and PC_71_BM showed the large aggregation domains. This may explain the low FF of the device based on P79. In contrast, the blend films of P80 and P81 with PC_71_BM showed a well-ordered separation network distribution between the polymer and acceptor (Figure 18d,e). When adding the solvent additive, a brillar-like bicontinuous interpenetrating network was observed in those films, which can explain the observed higher FF in the devices based P80 and P81 with solvent additives [129].

**Figure 19:**
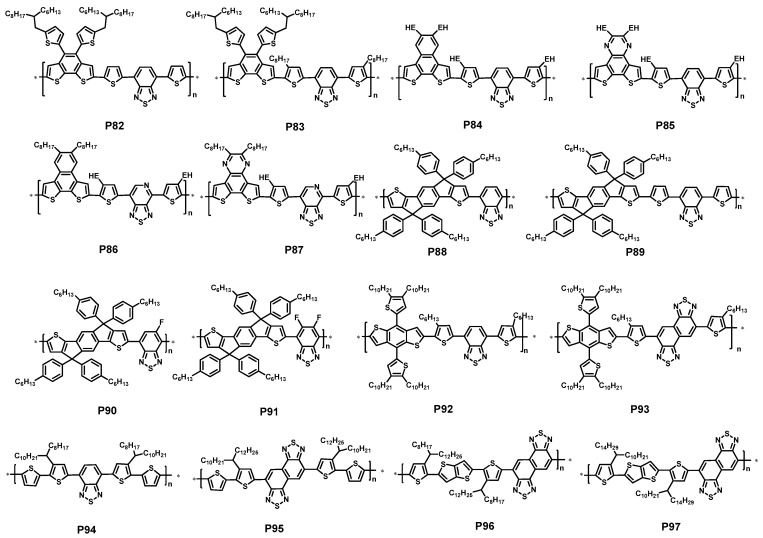
The chemical structures of 2,1,3-benzothiadiazole-based polymers for PSCs.

Beside the benzo[1,2-b:4,5-b′]dithiophene unit, benzo[2,1-b:3,4-b′]dithiophene, which has thiophenes units on the meta position of benzene unit, was also incorporated with 2,1,3-benzothiadiazole. The polymers, P82–P85 (Figure 19), were prepared via Stille polycondensation in the report of You and coworkers [130]. Because of a slightly longer conjugation length of benzo[2,1-b:3,4-b′]dithiophene unit and the electron-donating thiophene units, a slight decreased band-gaps of 1.63 eV and 1.69 eV for polymers P82 and P83 were found. The HOMO energy levels of P82 and P83 were −5.27 and −5.21 eV, respectively. Photovoltaic cells of P82/PC_61_BM and P83/PC_61_BM showed PCEs of 0.72% and 1.83%, respectively, with the *V*_oc_ of 0.55 and 0.77 V (Table 6). Low molecular weight (P82, *M*_n_ = 9 kDa) and limited solubility were responsible for the low performance of the P82-based device. Further, the group also reported another benzothiadiazole-based polymers P84 and P85 (Figure 19), which had naphtho[2,1-b:3,4-b′]dithiophene as donor unit in P84 and dithieno[3,2-f:2′,3′-h]quinoxaline as donor unit in P85, respectively [131]. The band-gaps of P84 and P85 were found to be 1.61 and 1.70 eV, respectively. The absorption spectra in both polymer thin films showed a more pronounced shoulder, indicating the strong π-stacking and polymer chain reorganization. P84 had a HOMO energy level of −5.34 eV, and the HOMO energy level of P85 was shifted to −5.46 eV due to the electron-deficient quinoxaline unit. Photovoltaic properties of P84 and P85 were investigated with a configuration of ITO/PEDOT:PSS/polymer:PC_61_BM/Ca/Al. The device measurement of P84/PC_61_BM (1:0.8, *w/w*) showed a PCE of 5.1% with a *V*_oc_ of 0.67 V, a *J*_sc_ of 14.20 mA/cm^2^ and a FF of 54% (Table 6). The high *J*_sc_ was believed to be related to the low band-gap of P84. In the case of P85/PC_61_BM (1:1.2, *w/w*), a *V*_oc_ of 0.83 V, a *J*_sc_ of 11.38 mA/cm^2^ and a FF of 45.6% were found, resulting in a PCE of 4.3% (Table 6). The higher *V*_oc_ was attributed to the lower-lying HOMO level of P85. Zhou et al. also reported another two polymers P86 and P87 containing thiadiazolo[3,4-*c*]pyridine [124]. The low band-gaps of P86 and P87 were calculated to be −1.53 and −1.56 eV from the absorption onset, respectively, which was 0.1–0.2 eV lower than the corresponding analogous benzothiadiazole-based polymers. The HOMO energy levels of P86 and P87 were −5.36 and −5.50 eV, respectively. The photovoltaic properties of these polymers were studied under the device structure of ITO/PEDOT:PSS/polymer:PC_61_BM/Ca/Al. It was encouraging that the P86/PC_61_BM-based device exhibited a maximum PCE of 6.20%, with a *V*_oc_ of 0.71 V, a *J*_sc_ of 14.16 mA/cm^2^ and a FF of 61.7%. The maximum PCE of 5.57% was found in the P87/PC_61_BM device, with a *V*_oc_ of 0.75 V, a *J*_sc_ of 13.49 mA/cm^2^ and a FF of 55.1% (Table 6).

More recently, Chen et al. and Liu et al. synthesized two alternating polymers (P88 and P89, Figure 19) that consisted of indacenodithiophene as electron-donating unit and 2,1,3-benzothiadiazole and 4,7-dithienyl-2,1,3-benzothiadiazole as the electron-accepting units. The band-gaps of P88 and P89 were 1.75 and 1.71 eV in thin films, respectively [132]. The HOMO energy levels of P88 and P89 were −5.35 and −5.19 eV, respectively. The FET hole mobility of P88 and P89 measured in the top-gate devices were 2 × 10^−2^ and 1.3 × 10^−2^ cm^2^·V^−1^·s^−1^, respectively. It was noted that the FET hole mobility of up to 1 cm^2^·V^−1^·s^−1^ in an analogous polymer of P88 had been reported in a top-gate bottom-contact device [110]. The photovoltaic cell of P88 gave a promising PCE of 6.1% with a *V*_oc_ of 0.85 V, a *J*_sc_ of 11.2 mA/cm^2^ and a FF of 67.2% (Table 6), showing a higher value than a random counterpart of P26 [133,134]. The device structure was ITO/PEDOT:PSS/P88:PC_71_BM/Ca/Al. Under the same condition, P89/PC_71_BM blend system had a *V*_oc_ of 0.78 V, a *J*_sc_ of 10.0 mA/cm^2^ and a FF of 55%, giving a PCE of 4.3% (Table 6). In addition, the inverted devices of P88 and P89 were also investigated with the device structure of ITO/ZnO/polymer:PC_71_BM (1:3, *w/w*)/PEDOT:PSS/Ag [135]. The devices showed PCEs of 4.7% and 4.1% for P88 and P89, respectively. It has been known that the introduction of silicon atoms into the polymer’s backbone will be able to bring several desirable characteristics to polymers, such as lower HOMO and LUMO levels, improved packing ability, and higher charge mobility.

**Figure 20:**
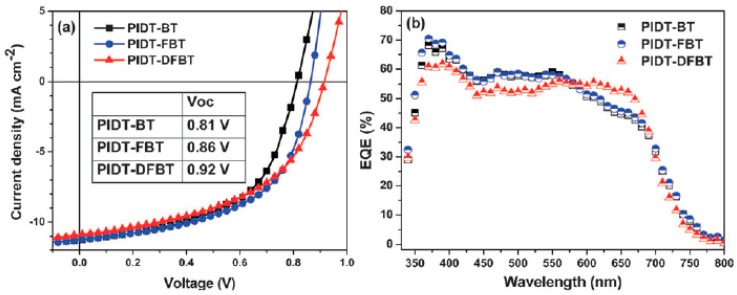
The *J*–*V* (**a**) and EQE (**b**) curves of PIDT–BT (P88), PIDT–FBT (P90) and PIDT–DFBT (P91)/PC_71_BM devices. Reproduced with permission from Reference [125], Copyright 2011, Royal Society of Chemistry.

To further elucidate the relationship upon the introduction of fluorine atom on *V*_oc_, Jen and coworkers synthesized two polymers P90 and P91 with mono- and di-fluoro-substituted benzothiadiazole as the acceptor and indacenodithiophene unit as the donor [125]. The investigation on optical properties found that the monofluoro-substituted polymer (P90) showed the same absorption and band-gap with that of non-fluoro-substituted polymer (P88). The absorption peak and onset of difluoro-substituted polymer (P91), however, showed a slight blue-shift compared with that of P90 and P88. The band-gaps were calculated to be 1.72 eV for P90 and 1.78 eV for P91. As indicated above, the fluorine atom would affect the HOMO energy level of the polymer. The CV measurements of P90 and P91 provided the clear evidence. The HOMO energy level of P90 was found to be −5.38 eV, along with 0.15 eV higher than that of P88. The HOMO energy level of P91 was further shifted to −5.48 eV. The lower-lying HOMO energy levels would be beneficial to *V*_oc_ in the corresponding BHJ cells. The photovoltaic properties of P90 and P91 were investigated with the device configuration of ITO/PEDOT:PSS/polymer:PC_71_BM/Ca/Al. The P90/PC_71_BM device showed an enhanced *V*_oc_ of 0.86 V, a *J*_sc_ of 11.23 mA/cm^2^ and a FF of 56%, with the PCE of 5.40% (Table 6). Under the same condition, a *V*_oc_ of 0.81 V, a *J*_sc_ of 11.23 mA/cm^2^ and a FF of 55% with a PCE of 5.02% was achieved for P91 device (Table 6). It was found that the improved PCE in P90 device mainly came from the enhanced *V*_oc_ as the result of lower-lying HOMO energy level. In P91/PC_71_BM device, a *V*_oc_ of as high as 0.92 V was reached with an overall PCE of 5.10%. The overall performance in P91 device was offset by the loss of *J*_sc_ and FF (Figure 20a). The EQE curves of P88 and P90 devices were almost overlapped. This was consistent with the similarities in the measured *J*_sc_ and FF in both devices. Whereas in the P91 device, the low EQE value between 350 to 550 nm coming from the PC_71_BM may be one of the main factors for the measured low *J*_sc_ (Figure 20b). Further optimization will be possible to push a higher value for both P90 and P91 based BHJ cells. Overall, these results clearly showed that fine tuning on the HOMO energy level to achieve the increased *V*_oc_ in BHJ cells was an effective way to improve the overall performance.

**Figure 21:**
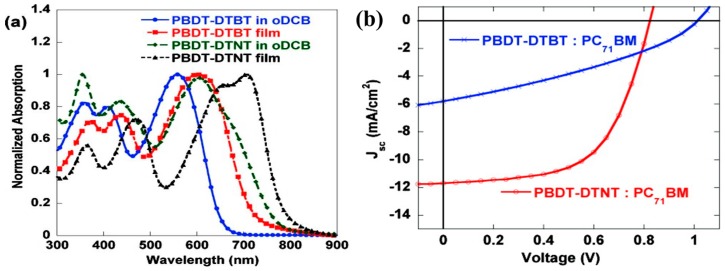
(**a**) Normalized absorption spectra of PBDT-DTNT(P93) and PBDT-DTBT(P92) in *o*-DCB solutions and in films; (**b**) *J*–*V* curves of the PBDT-DTBT(P92)/PC_71_BM and PBDTDTNT(P93)/PC_71_BM devices. Reproduced with permission from Reference [136], Copyright 2011, American Chemical Society.

Naphtho[1,2-c:5,6-c]bis[1,2,5]thiadiazole (NT) unit is a very useful acceptor unit, which contains two fused 1,2,5-thiadiazole rings that could lower the band gap, enhance the interchain packing, and improve the charge mobility of polymer. In 2011, Wang et al. used NT unit as acceptor unit with BDT as donor unit to synthesize the new polymer P93. As shown in Figure 21a, P93 clearly exhibited more obvious red-shifted absorption spectra both in *o*-dichlorobenzne (*o*-DCB) solution and thin film, which was likely a result of the more electron deficient nature of the NT acceptor. By changing the acceptor from BT to NT, the optical band gaps of the polymers were decreased from 1.73 eV for PBDT-DTBT(P92) to 1.58 eV for PBDT-DTNT(P93), the latter being much closer to the ideal band gap for PSC donor materials. When fabricated solar devices with PC_71_BM, P93 showed a better PCE of 6.00% with *V*_oc_ of 0.80 V, a *J*_sc_ of 11.71 mA/cm^2^ and a FF of 61%. A slightly low PCE of 2.11% was obtained for the P92 at the same condition, which was ascribed to its low *J*_sc_ and FF (Table 6) (Figure 21b) [136].

**Figure 22:**
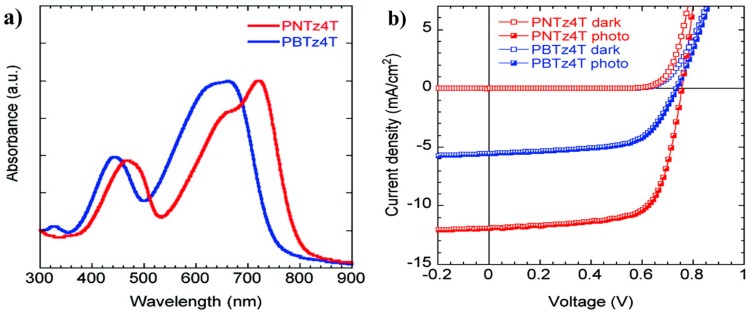
(**a**) The UV-vis absorption spectra of PNTz4T(P95) and PBTz4T(P94) in the thin film; (**b**) *J*−*V* curves of BHJ solar cells (ITO/PEDOT:PSS/ PNTz4T(P95) and PBTz4T(P94):PC_61_BM/LiF/Al); polymer:PC61BM = 1:1.5 for PNTz4T and 1:1 for PBTz4T. Reproduced with permission from Reference [137], Copyright 2012, American Chemical Society.

Later, Osaka et al. also used the NT unit as an acceptor with quaterthiophene as a donor unit to develop the polymer P95. In the film state, owning to the highly π-extended structure, P95 showed a much more red-shifted absorption than P94 (Figure 22a). At the same time, a higher hole mobility of 0.35 cm^2^·V^−1^·s^−1^ was got for P95. Finally, when solar cells were fabricated with PC_61_BM, P95 obtained a nice PCE of 6.3% with *V*_oc_ of 0.76 V, a *J*_sc_ of 12.0 mA/cm^2^ and a FF of 69%. A slightly low PCE of 4.4% was achieved for P94 (Table 6) (Figure 22b) [137].

**Figure 23:**
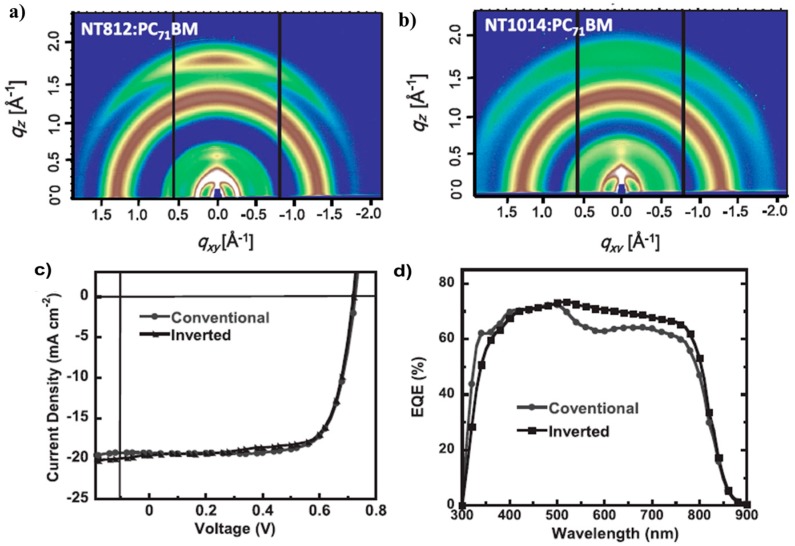
(**a**) Grazing incidence X-ray diffraction patterns of NT812 (P96):PC_71_BM; (**b**) Grazing incidence X-ray diffraction patterns of NT1014 (P97):PC_71_BM; (**c**) *J*–*V* characteristics; (**d**) external quantum efficiency spectra for conventional and inverted devices based on NT812(P96). Reproduced with permission from Reference [138], Copyright 2016, Wiley.

Huang and coworkers further copolymerized NT unit with 2,5-bis(3-alkylthiophen-2-yl)thieno[3,2-*b*]thiophene to make two polymers, P96 and P97, which have different alkyl substitutes. It was interesting that P97 showed a red-shifted than that of P96 even they had the same polymer backbone, which was believed to be responsible for the different π–π stacking behavior. As shown in Figure 23a,b, the ill-packing of the polymer chain and less-preferential crystal orientations were found for both polymers. The photovoltaic cells based on P96 showed the PCEs of 10.33% and 10.23% in the inverted and conventional constructions, respectively, with the *J*_sc_ of as high as 19.94 mA/cm^2^ (Table 6), which was ascribed to the enhanced EQE in the range from 500 to 900 nm (Figure 23c,d) [138].

### 2.4. Polymers with Nonlinear Optical Chromophores

Commonly, conjugated polymers for PSCs have the alternating electron-rich and electron-deficient units along their backbone, which could tune the absorption spectra and energy levels through selecting the appropriated donor and acceptor units.

**Figure 24:**
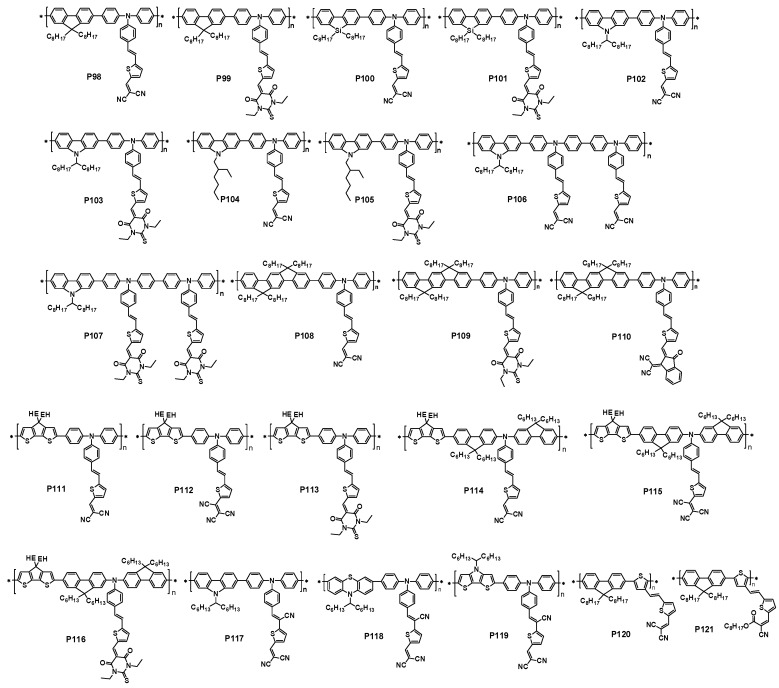
The chemical structures of polymers with the nonlinear optical chromophores.

**Figure 25:**
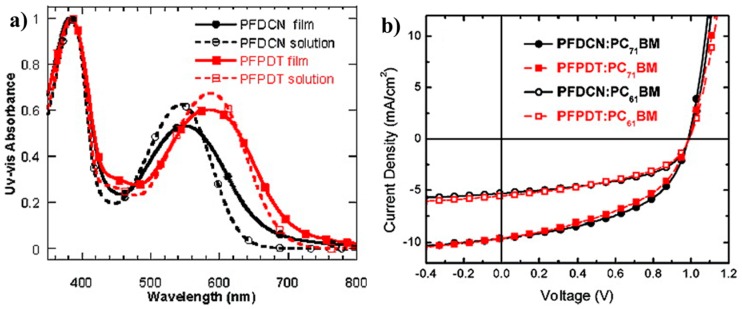
(**a**) *J*–*V* curves of PFDCN (P98) and PFPDT (P99)-based solar cells under AM 1.5G illumination; (**b**) External quantum efficiency spectrum of PFDCN (P98) and PFPDT (P99)-based solar cells. Reproduced with permission from Reference [139], Copyright 2009, American Chemical Society.

In contrast with these alternating donor-acceptor conjugated polymers, Jen and coworkers first reported two representative polymers (P98 and P99, Figure 24), in which the polymers had the electron-rich polymer main chain and the acceptor are locating at the end of polymer side chains through the conjugated styrylthiophene π-bridge [139]. This new-type of polymers had the advantages of well-established knowledge of nonlinear optical chromophores. With different chromophores, the absorption spectra and energy levels of the resultant polymers could be tuned. In the P98 and P99 structure, a fluorene-triphenylamine copolymer was selected as the polymer backbone because of the high hole mobility. The nonlinear optical chromophores of dicyanovinyl (DCN) and 1,3-diethyl-2-thiobarbituric acid (DTA) were used as the acceptors in the side chains of P98 and P99, respectively. In the absorption spectrum, two obvious absorption peaks were observed for P98 and P99, in which the peaks in the short wavelengths were corresponding to the π–π* transition of their conjugated main chains and the others were ascribed to the strong ICT characters of the polymer side chains. The optical band-gap of P98 was 1.87 eV in thin film (Figure 25a). It was reduced to 1.76 eV in P99 when a stronger acceptor was used. The HOMO energy levels of P98 and P99 were found to be −5.30 and −5.26 eV. The LUMO levels of P98 and P99, calculated from their optical band gaps and HOMO energy levels, were −3.43 and −3.50 eV. It was clear that both of the polymers exhibited similar HOMO energy levels because of the same polymer main chain, while the LUMO energy levels were dominated by the acceptors on the side chains. The photovoltaic properties of P98 and P99 were investigated in a conventional device configuration of ITO/PEDOT:PSS/polymer:PCBM (1:4, *w/w*)/Ca/Al. In the P98/PC_61_BM device, a PCE of 2.55% was achieved with a *V*_oc_ of 0.99 V, a *J*_sc_ of 5.12 mA/cm^2^ and a FF of 50%. A similar performance was reported in the P99/PC_61_BM blend system. However, when PC_71_BM was applied as the electron acceptor in BHJ cells, the PCE of P98/PC_71_BM device was improved up to 4.74% with a *V*_oc_ of 0.99 V, a *J*_sc_ of 9.62 mA/cm^2^ and a FF of 50% (Table 7). The PCE of up to 4.37% in P99/PC_71_BM device was observed (Figure 25b). The increased performance with PC_71_BM was believed to be the enhanced absorption in the range of 440 to 530 nm. It was noted that the *V*_oc_ of as high as 0.99 V was reached because of the low-lying HOMO energy level. In addition, the SCLC hole mobility of 5.27 × 10^−4^ cm^2^·V^−1^·s^−1^ for P98 and 1.16 × 10^−3^ cm^2^·V^−1^·s^−1^ for P99 were found and were beneficial for the highly efficient PSCs.

It is well known that the bridging atom on the polymer main chain would give significant influence on the properties of the polymer for PSCs [75,99,102,133,140]. Recently, the Si- and N-analogous polymers of P98 and P99 were also synthesized by several groups. Polymers P100 and P101 (Figure 25) developed by Cao and coworkers showed the similar band-gaps with P98 and P99, but a slight higher HOMO energy level (−5.32 eV for P100, −5.35 eV for P101) than that of P98 and P99 [141]. The photovoltaic measurements of P100 and P101-based devices in the configuration of ITO/PEDOT:PSS/polymer:PC_71_BM (1:4, *w/w*)/Ba/Al exhibited lower performance than that of P98 and P99. The device of P100 gave a *V*_oc_ of 0.85 V, a *J*_sc_ of 6.69 mA·cm^−2^ and a FF of 36.9%, with an overall PCE of 2.50%. A better performance of PCE of 3.15% with a *V*_oc_ of 0.90 V, a *J*_sc_ of 6.22 mA/cm^2^ and a FF of 45.1% (Table 7) was found in the case of P101 because of the more uniform morphology in P101/PC_71_BM blend film than that of P100. The morphology difference of P100 and P101 blend films were responsible for different processing solvents. For the carbazole-based polymers, it was interesting that polymer P102 and P103 (Figure 25) were reported by Duan et al., Zhang et al. and Hsu et al. at almost same time [141,142,143]. The band-gaps of P102 and P103 were around 1.70–1.80 eV. The HOMO level of −5.30 eV for both polymers was reported. Photovoltaic tests of a P102/PC_71_BM and P103/PC_71_BM blend system exhibited a PCE of 2%–3%, with various *V*_oc_ due to the different cathode used in these devices (Table 7). In addition, another two polymers, P104 and P105, which have 2-ethylhexyl side chain in carbazole unit compared with the 2-octylnonyl side chains of P102 and P103, were also synthesized by Duan et al. [144]. The BHJ cells of P104 and P105 under the same conditions showed an improved performance compared with P102 and P103. The PCE of 4.16% with a *V*_oc_ of 0.91 V, a *J*_sc_ of 8.94 mA/cm^2^ and a FF of 51% was reported for P104 device. The PCE of P105 device was up to 3.52% (Table 7). The improved photovoltaic performances in P104 and P105 devices were attributed to the less bulky in polymer side chains. Further, to enhance the absorption coefficient of these polymers, polymers P106 and P107 that had two nonlinear optical chromophores in one repeat unit were also prepared (Figure 25) [144]. Unfortunately, there were no increases in the photovoltaic performance found in P106/PC_71_BM and P107/PC_71_BM blend systems with the same device structures.

Indenofluorene, a donor unit containing two linearly overlapped fluorenes, was also copolymerized with these NLO chromophores aiming to achieve broader and stronger absorption than that of fluorene counterpart as the result of extended pi-conjugation in indenofluorene unit. The polymers, P108–P110, were synthesized with a similar method as P98–P107 [145]. The band-gaps were tuned from 1.86 eV in P108 to 1.53 eV in P110 by selecting stronger chromophore. The HOMO energy levels of three polymers were similar to be around −5.32 eV with various LUMO levels from −3.46 to −3.79 eV. The photovoltaic cells with the structure of ITO/PEDOT:PSS/P108–P110:PC_71_BM (1:4 wt)/Ca/Al were fabricated. The highest PCE was achieved to be 3.1% for P108 with a *V*_oc_ of 0.93 V, a *J*_sc_ of 7.4 mA/cm^2^ and a FF of 44%. The PCEs of P109 and P110 were 2.85% and 2.6% with similar parameters (Table 7).

Beside fluorene, silafluorene, carbazole and indenofluorene units, the cyclopentadithiophene as the donor unit was also incorporated into these D-π-A type polymers. A series of polymers, P111-P116, were synthesized via through Stille polycondensation between cyclopentadithiophene’s distannyl compound and the appropriated bromide moiety (Figure 25) [146]. In these polymers, three different acceptor units, DCN, DTA and tricyanovinyl (TCN), were selected with the acceptor strength of TCN > DTA > DCN. In addition, a new donor moiety of difluorenylphenyl amino in polymer main chain was also used. The optical band-gaps of these polymers followed in the trend of acceptor strength, 1.70 and 1.80 eV for P111 and P114, 1.69 eV for P113 and P116, and 1.34 eV for P112 and P115. The HOMO energy levels of triphenylamine-based polymers were similar at around −5.06 eV. In the difluorenylphenyl amino-based polymers, the HOMO energy levels moved to −5.20 eV. The photovoltaic performance of these polymers in the device structure of ITO/PEDOT:PSS/polymer:PC_71_BM(1:4, *w/w*)/Ca/Al showed the PCE between 0.20% and 1.40% (Table 7). The *V*_oc_ of P111-P113 devices were 0.58–0.64 V, and this value was increased to 0.70 V in P114–P116 devices because of the lower-lying HOMO energy level. It shows an effective way to tune the *V*_oc_ of PSCs by selecting the stronger donor moiety. Recently, Lin and coworkers further reported three so-called D-π-A type polymers (P115–P117, Figure 25) by choosing carbazole, phenothiazine, and dithienopyrole as the donor unit of polymer main chain. In addition, a cyano unit was also attached into the vinyl bond between triphenylamine and thiophene spacer to further enhance the absorption spectra coverage of resulting polymers [147]. The optical band-gaps of P117–P119 were between 1.66 and 1.72 eV. The HOMO energy levels were increased following the strength of donor units, P119 (−5.12 eV) > P118 (−5.20 eV) > P117 (−5.32 eV). The relatively low PCEs of 0.34%–1.01% in P115–P117 devices were reported with a *V*_oc_ of 0.63–0.79 V in the device configuration of ITO/PEDOT:PSS/P117–P119:PC_61_BM (1:4, *w/w*)/Ca/Al (Table 7). More recently, the poly (fluorene-*co*-thiophene) polymers (P118 and P119, Figure 25) were also prepared with conjugated pendant side of dicyanovinyl and acetate cyanovinyl by Zhan and coworkers [148]. A lower-lying HOMO level of −5.51 eV was observed in both P120 and P121. The photovoltaic measurement showed the PCEs of 0.48% and 1.33% for P120 and P121, respectively (Table 7). Although the poor performances were observed, it is necessary to note that the optimized weight ratio of polymer to PC_61_BM was 1:1 and 1:2, respectively, for P120 and P121, instead of the optimized 1:4 ratio of polymer to PCBM in the most D-π-A type polymers discussed above.

## 3. Summaries and Outlooks

In summary, the recent developments in conjugated polymers and fullerene derivatives for the high efficient and stable PSCs have been reviewed. In the polymer part, a PCE of more than 10% has been demonstrated and provides a promising candidate for the commercialization of PSCs. In addition, some new designs, such as D-π-A side chains polymer, also show the significant potential to reach higher performance through the rational polymer design. In the development of high efficient PSCs, the rules are becoming clear in the past decades from the materials development point of view. First and important, the conjugated polymers should have a low band-gap (1.2–1.9 eV) and strong absorption co-efficient, which will potentially lead to high *J*_sc_. In addition, the polymer should possess the low-lying HOMO energy level for high *V*_oc_, but the offset of more than 0.3–0.6 eV between the LUMO energy levels of the polymer and acceptor in BHJ layer should also be considered for ensuring the efficient charge separation. So, there is a dilemma such that higher *V*_oc_ and lower band-gap commonly lead to a lower-lying LUMO energy level, and thus inefficient charge separation. The introduction of an electron-withdrawing group (not limited to the fluorine atom) provides an alternative method to overcome this dilemma. On the other hand, the Zhang group and Wu group both reported that the steric hindrance that exists in the polymer may be an important factor influencing the *V*_oc_ of devices [149,150]. As a result, low band-gap of polymers without sacrificing efficient charge separation as well as high *V*_oc_ will become available for achieving highly efficient PSCs. It can be expected that more and more future work will follow this direction. Other basic requirements for the development of polymers are: good hole mobility and good solubility in common organic solvents. In the fullerene derivatives for efficient PSCs, a high-lying LUMO energy level is important for offering high *V*_oc_ in PSC. In addition, good electron mobility is a basic requirement for these developed and developing fullerene derivatives for the efficient charge transportation. Another important thing that needs to be considered is that the higher LUMO energy level of fullerene will diminish the offset with polymer’s LUMO energy level, making it less than the requirement for charge separation. Another aspect which should be paid more attention is the green chemistry in the polymer synthesis. Even though the development of this methodology fall far behind the conventional method to synthesize the polymer (i.e., the Suzuki or Stille methodologies, which both use poisonous materials), the green chemistry methodology, which can be run with low energy intensity, minimal production of toxic waste, and low cost, will be a promising technology to apply in the conjugated polymer synthesis in the future for the sustainable development of humankind. Overall, the BHJ cell with the active layer that follows all these rules will be expected to show a promised PCE of more than 15%, the next target for the PSC society.

## Figures and Tables

**Table 1:** Optical, electronic, and photovoltaic performances of P1–P24.

Polymer	*E*_g_^opt^ (eV)	HOMO (eV)	*V*_OC_ (V)	*J*_SC_ (mA/cm^2^)	FF (%)	PCE (%)	Reference
P1	1.62	−4.90	0.58	12.5	65.4	4.76	[43]
P2	1.59	−4.94	0.60	12.8	66.3	5.10	[44]
P3	1.60	−5.04	0.74	13.1	56.8	5.53	[44]
P4	1.63	−5.12	0.76	9.20	44.5	3.10	[44]
P5	1.62	−5.01	0.68	10.3	43.1	3.02	[44]
P6	1.61	−5.01	0.62	7.74	47.0	2.26	[44]
P7	-	−5.15	0.76	13.6	54.5	5.63	[45]
P7	1.84	−5.15	0.75	17.5	70.0	9.20	[48]
P8	1.75	−5.41	0.68	11.1	42.2	3.20	[49]
P9	1.73	−5.48	0.75	9.10	39.4	2.70	[49]
P10	1.61	−5.12	0.70	14.7	64.0	6.58	[50]
P11	1.61	−5.22	0.76	15.2	66.9	7.73	[51]
P12	1.60	−5.10	0.68	14.6	62.6	6.21	[52]
P13	1.60	−5.10	0.74	17.8	58.7	7.59	[53]
P14	1.58	−5.22	0.80	15.7	74.3	9.35	[54]
P14	1.59	−5.22	0.82	17.5	72.0	10.3	[55]
P15	1.51	−5.33	0.80	17.5	67.9	9.48	[56]
P16	1.62	−5.45	0.86	16.4	62.2	9.00	[57]
P17	1.65	−5.12	0.76	14.1	58.0	6.22	[58]
P18	1.59	−5.21	0.73	16.6	64.1	7.79	[59]
P19	1.67	−5.24	0.89	13.0	65.3	7.60	[60]
P20	1.68	−5.30	0.88	10.7	52.1	4.90	[60]
P21	1.76	−5.04	0.66	15.0	58.0	5.62	[61]
P22	1.77	−5.08	0.62	9.60	50.0	2.98	[61]
P23	1.59	−5.12	0.69	16.4	66.3	7.81	[62]
P24	1.64	−4.90	0.78	15.2	72.3	8.60	[63]

**Table 2:** Optical, electronic, and photovoltaic performances of P25–P44.

Polymer	*E*_g_^opt^ (eV)	HOMO (eV)	*V*_OC_ (V)	*J*_SC_ (mA/cm^2^)	FF (%)	PCE (%)	Reference
P25	1.82	−5.40	0.85	9.80	66.0	5.50	[70]
P26	1.75	−5.48	0.87	8.10	56.0	3.90	[72]
P27	1.70	−5.57	0.86	10.1	68.0	6.30	[72]
P28	2.20	−5.50	0.76	3.90	42.0	1.23	[73]
P29	1.77	−5.61	0.96	6.52	54.0	3.42	[74]
P30	1.67	−5.43	0.80	10.0	47.0	3.74	[75]
P31	1.70	−5.44	0.91	2.32	56.0	1.18	[75]
P31	1.73	−5.57	0.88	12.2	68.0	7.30	[77]
P32	1.59	−5.11	0.66	4.98	50.0	1.65	[76]
P33	1.69	−5.60	0.85	12.6	68.0	7.30	[78]
P34	-	-	0.90	9.65	61.0	5.30	[79]
P35	-	-	0.90	10.9	65.0	6.40	[79]
P36	1.84	−5.42	0.93	6.58	56.0	3.42	[80]
P37	1.84	−5.44	0.91	10.3	51.0	4.79	[80]
P38	1.85	−5.61	1.00	9.79	63.0	6.17	[81]
P39	1.79	−5.41	1.05	10.6	60.0	6.70	[82]
P40	1.88	−5.36	0.87	12.2	62.0	6.60	[83]
P41	1.82	−5.56	0.95	8.02	62.0	4.70	[84]
P42	1.50	−4.85	0.41	7.39	48.0	1.44	[85]
P43	-	−5.66	0.94	10.2	61.0	5.77	[86]
P44	-	−5.49	0.88	10.7	62.0	5.80	[87]

**Table 3:** Optical, electronic, and photovoltaic performances of P45–P50.

Polymer	*E*_g_^opt^ (eV)	HOMO (eV)	*V*_OC_ (V)	*J*_SC_ (mA/cm^2^)	FF (%)	PCE (%)	Reference
P45	1.84	−5.42	0.94	5.72	43.3	2.32	[88]
P46	1.75	−5.55	0.96	11.2	51.4	5.52	[88]
P47	1.65	−5.49	0.76	2.90	43.0	0.95	[89]
P48	1.86	−5.56	0.89	7.60	57.0	3.90	[89]
P49	1.83	−5.66	0.66	1.20	26.0	0.20	[89]
P50	1.83	−5.35	0.83	5.28	61.0	2.68	[90]

**Table 4:** Optical, electronic, and photovoltaic performances of P51–P64.

Polymer	*E*_g_^opt^ (eV)	HOMO (eV)	*V*_OC_ (V)	*J*_SC_ (mA/cm^2^)	FF (%)	PCE (%)	Reference
P51	-----	−5.50	0.88	10.6	66.0	6.10	[108]
P52	1.59	−5.18	0.72	12.3	70.5	6.26	[109]
P53	1.68	−5.24	0.82	13.3	56.9	6.17	[112]
P54	1.66	−5.27	0.83	5.86	33.2	1.61	[114]
P55	1.60	−5.22	0.69	13.9	61.8	5.83	[114]
P56	1.80	−5.32	0.81	8.86	48.8	3.49	[115]
P57	1.70	−5.25	0.88	9.39	52.0	4.30	[116]
P58	1.62	−5.36	0.76	16.2	62.1	7.64	[117]
P59	1.65	−5.54	0.78	12.2	67.5	6.41	[118]
P60	1.72	−5.29	0.81	10.4	61.0	5.08	[119]
P61	1.72	−5.35	0.81	10.2	62.0	5.11	[119]
P62	1.72	−5.45	0.86	11.4	74.0	7.26	[119]
P63	1.66	−5.50	0.76	16.5	71.0	8.80	[120]
P64	1.80	−5.70	0.93	13.1	74.0	9.00	[120]

**Table 5:** Optical, electronic, and photovoltaic performances of P65–P81.

Polymer	*E*_g_^opt^ (eV)	HOMO (eV)	*V*_OC_ (V)	*J*_SC_ (mA/cm^2^)	FF (%)	PCE (%)	Reference
P65	1.70	−5.33	0.83	7.70	59.6	3.85	[121]
P66	1.70	−5.26	0.81	9.70	54.8	4.31	[121]
P67	1.51	−5.47	0.85	12.8	58.2	6.32	[122]
P68	1.56	−5.48	0.69	8.89	55.4	3.40	[123]
P69	1.60	−5.30	0.69	6.15	44.3	1.88	[123]
P70	1.70	−5.40	0.87	10.0	57.3	5.00	[124]
P71	1.70	−5.54	0.91	12.9	61.2	7.20	[124]
P72	1.70	−5.31	0.78	15.4	69.2	8.30	[126]
P73	1.76	−5.34	0.76	13.2	61.9	6.20	[126]
P74	1.72	−5.33	0.68	11.9	55.2	4.46	[126]
P75	1.70	−5.35	0.88	12.9	70.9	8.07	[127]
P76	1.75	−5.31	0.92	10.7	57.5	5.66	[105]
P77	1.78	−5.40	0.92	11.6	65.0	6.92	[128]
P78	1.80	−5.48	0.95	12.0	64.0	7.27	[128]
P79	1.75	−5.50	0.84	11.8	49.4	4.91	[129]
P80	1.82	−5.63	0.94	12.5	69.0	8.10	[129]
P81	1.81	−5.56	0.93	13.0	74.5	9.02	[129]

**Table 6:** Optical, electronic, and photovoltaic performances of P82–P97.

Polymer	*E*_g_^opt^ (eV)	HOMO (eV)	*V*_OC_ (V)	*J*_SC_ (mA/cm^2^)	FF (%)	PCE (%)	Reference
P82	1.63	−5.27	0.55	3.53	36.8	0.72	[130]
P83	1.69	−5.21	0.67	6.38	50.8	1.83	[130]
P84	1.61	−5.34	0.67	14.2	54.0	5.10	[131]
P85	1.70	−5.46	0.83	11.4	45.6	4.30	[131]
P86	1.53	−5.36	0.71	14.2	61.7	6.20	[124]
P87	1.56	−5.50	0.75	13.5	55.1	5.57	[124]
P88	1.75	−5.35	0.85	11.2	67.2	6.10	[133]
P89	1.71	−5.19	0.78	10.0	55.0	4.30	[133]
P90	1.72	−5.38	0.86	11.2	56.0	5.40	[125]
P91	1.78	−5.48	0.81	11.2	55.0	5.02	[125]
P92	1.73	−5.26	1.00	5.80	34.6	2.11	[136]
P93	1.58	−5.19	0.80	11.7	61.0	6.00	[136]
P94	1.65	−5.07	0.74	8.70	71.0	4.40	[137]
P95	1.54	−5.16	0.76	12.0	69.0	6.30	[137]
P96	1.40	−5.29	0.72	19.9	71.5	10.2	[138]
P97	1.40	−5.29	0.77	7.25	52.9	3.03	[138]

**Table 7:** Optical, electronic, and photovoltaic performances of P98–P121.

Polymer	*E*_g_^opt^ (eV)	HOMO (eV)	*V*_OC_ (V)	*J*_SC_ (mA/cm^2^)	FF (%)	PCE (%)	Reference
P98	1.87	−5.30	0.99	9.62	50.0	4.74	[139]
P99	1.76	−5.26	0.99	9.61	46.0	4.37	[139]
P100	1.83	−5.32	0.85	6.69	36.9	2.50	[141]
P101	1.74	−5.35	0.90	6.22	45.1	3.15	[141]
P102	1.88	−5.29	0.91	7.52	44.0	2.91	[143]
P103	1.77	−5.30	0.89	6.23	38.0	2.09	[143]
P104	1.83	−5.23	0.91	8.94	51.0	4.16	[144]
P105	1.74	−5.25	0.92	8.18	47.0	3.52	[144]
P106	1.83	−5.20	0.83	7.18	39.0	2.34	[144]
P107	1.74	−5.24	0.84	6.53	40.0	2.19	[144]
P108	1.86	−5.32	0.93	7.40	44.0	3.10	[145]
P109	1.76	−5.36	0.93	7.30	41.0	2.80	[145]
P110	1.53	−5.32	0.88	7.30	40.0	2.60	[145]
P111	1.70	−5.08	0.64	4.45	40.0	1.14	[146]
P112	1.69	−5.06	0.62	4.65	41.0	1.18	[146]
P113	1.34	−5.06	0.58	1.26	32.0	0.22	[146]
P114	1.80	−5.20	0.79	3.51	49.0	1.36	[146]
P115	1.69	−5.21	0.70	4.57	43.0	1.38	[146]
P116	1.34	−5.17	0.70	1.17	38.0	0.31	[146]
P117	1.66	−5.32	0.79	3.99	32.0	1.01	[147]
P118	1.70	−5.20	0.65	1.91	30.6	0.38	[147]
P119	1.72	−5.12	0.63	1.78	30.3	0.34	[147]
P120	-	−5.51	0.80	1.45	41.0	0.48	[148]
P121	-	−5.51	0.90	4.33	34.0	1.33	[148]
